# In vivo biodistribution and physiologically based pharmacokinetic modeling of inhaled fresh and aged cerium oxide nanoparticles in rats

**DOI:** 10.1186/s12989-016-0156-2

**Published:** 2016-08-20

**Authors:** Dingsheng Li, Masako Morishita, James G. Wagner, Mohammad Fatouraie, Margaret Wooldridge, W. Ethan Eagle, James Barres, Ulrika Carlander, Claude Emond, Olivier Jolliet

**Affiliations:** 1 Department of Environmental Health Sciences, University of Michigan, Ann Arbor, MI 48109 USA; 2 Department of Pathobiology and Diagnostic Investigation, Michigan State University, East Lansing, MI 48824 USA; 3 Department of Mechanical Engineering, University of Michigan, Ann Arbor, MI 48109 USA; 4 Institute of Environmental Medicine, Karolinska Institutet, Stockholm, SE-171 77 Sweden; 5 BioSimulation Consulting Inc., Newark, DE 19713 USA

**Keywords:** Cerium oxide nanoparticles, Inhalation, Biodistribution, PBPK modeling

## Abstract

**Background:**

Cerium oxide (CeO_2_) nanoparticles used as a diesel fuel additive can be emitted into the ambient air leading to human inhalation. Although biological studies have shown CeO_2_ nanoparticles can cause adverse health effects, the extent of the biodistribution of CeO_2_ nanoparticles through inhalation has not been well characterized. Furthermore, freshly emitted CeO_2_ nanoparticles can undergo an aging process by interaction with other ambient airborne pollutants that may influence the biodistribution after inhalation. Therefore, understanding the pharmacokinetic of newly-generated and atmospherically-aged CeO_2_ nanoparticles is needed to assess the risks to human health.

**Methods:**

A novel experimental system was designed to integrate the generation, aging, and inhalation exposure of Sprague Dawley rats to combustion-generated CeO_2_ nanoparticles (25 and 90 nm bimodal distribution). Aging was done in a chamber representing typical ambient urban air conditions with UV lights. Following a single 4-hour nose-only exposure to freshly emitted or aged CeO_2_ for 15 min, 24 h, and 7 days, ICP-MS detection of Ce in the blood, lungs, gastrointestinal tract, liver, spleen, kidneys, heart, brain, olfactory bulb, urine, and feces were analyzed with a mass balance approach to gain an overarching understanding of the distribution. A physiologically based pharmacokinetic (PBPK) model that includes mucociliary clearance, phagocytosis, and entry into the systemic circulation by alveolar wall penetration was developed to predict the biodistribution kinetic of the inhaled CeO_2_ nanoparticles.

**Results:**

Cerium was predominantly recovered in the lungs and feces, with extrapulmonary organs contributing less than 4 % to the recovery rate at 24 h post exposure. No significant differences in biodistribution patterns were found between fresh and aged CeO_2_ nanoparticles. The PBPK model predicted the biodistribution well and identified phagocytizing cells in the pulmonary region accountable for most of the nanoparticles not eliminated by feces.

**Conclusions:**

The biodistribution of fresh and aged CeO_2_ nanoparticles followed the same patterns, with the highest amounts recovered in the feces and lungs. The slow decrease of nanoparticle concentrations in the lungs can be explained by clearance to the gastrointestinal tract and then to the feces. The PBPK model successfully predicted the kinetic of CeO_2_ nanoparticles in various organs measured in this study and suggested most of the nanoparticles were captured by phagocytizing cells.

**Electronic supplementary material:**

The online version of this article (doi:10.1186/s12989-016-0156-2) contains supplementary material, which is available to authorized users.

## Background

Engineered nanoparticles have emerged as innovative materials that hold multiple promising features when compared to their bulk counterparts [1]. Industrial, commercial, medical and consumer applications of nanomaterials have raised concerns about the potential human health impacts, given toxic effects of nanoparticles in animal studies [2–5]. This paper focuses on inhalation exposure and the systemic distribution of cerium oxide (CeO_2_) nanoparticles, which are mainly used as a diesel fuel additive to reduce particulate emissions [6, 7] and can be released to the environment by diesel engines. Several in vitro and in vivo studies have shown CeO_2_ nanoparticles can generate reactive oxygen species that induce oxidative stress and inflammatory responses [8, 9]. To better understand the toxic effects of CeO_2_ nanoparticles, it is necessary to first characterize the biodistribution of CeO_2_ nanoparticles in the entire body.

Several studies have shown intravenously injected CeO_2_ nanoparticles in rats could be found in all major organs up to 90 days after injection [10–12]. An intratracheal instillation study found that besides the lungs, the largest amount of instilled CeO_2_ nanoparticles was in the feces in the first 2 days after exposure [13]. Recently conducted inhalation studies on CeO_2_ nanoparticles showed the lungs are the major deposit organ while CeO_2_ nanoparticles could penetrate from the lungs into the systemic circulation as they were found in other body organs [8, 14]. However, these inhalation studies did not include feces and failed to provide a more comprehensive mass balance of the inhaled dose of CeO_2_ nanoparticles. In addition, diesel fuel combustion emits CeO_2_ nanoparticles directly into the air where the particles can interact with reactive gases found in the urban air environment. There is therefore a need to characterize the environmental transformation and physicochemical properties of aged CeO_2_ nanoparticles accounting for the interactions with UV radiation and the typical reactive gases found in ambient air, and to compare the biodistribution of aged CeO_2_ nanoparticles with the biodistribution of freshly-combusted CeO_2_ nanoparticles.

To better understand exposure, provide insights on the main mechanisms responsible for biodistribution and possibly extrapolate biobehavior from rodent models to humans, it is useful to complement biodistribution experimental data with pharmacokinetic modeling. Physiologically based pharmacokinetic (PBPK) models have been used to study and predict the biodistribution of chemicals inside the body for decades [15]. Several PBPK models have been specifically developed for nanoparticles [16–19] but failed to specifically address the role of phagocytosis, which has been observed as a key process of nanoparticle biodistribution [20–22]. Li et al. [23] built a whole-body PBPK model that incorporated phagocytosis, but the model was limited to intravenous injection of nanoparticles. Kolanjiyil and Kleinstreuer [24] developed a multicompartment model for inhaled nanoparticles, but the model was based on relatively simple transfer rates between each compartment and therefore lacked physiological significance. PBPK models for inhalation exposure of silver nanoparticles and for instillation of gold nanoparticles have been developed by Bachler et al. [25, 26]. These models have one compartment for the respiratory system and the latter lack translocation to the gastrointestinal tract via mucus escalator, which has been indicated as an important elimination pathway for cerium nanoparticles [13]. In the PBPK model by Sweeney and colleagues [27], the researchers extended a previous lung model to include systemic exposure of silver, titanium dioxide and iridium nanoparticles. The model divided the respiratory system into three compartments and allowed uptake to the olfactory system via the nose and in tissue nanoparticles could be quasi-irreversibly sequestered and cleared to feces. Currently, there is no detailed PBPK model describing the biodistribution of inhaled CeO_2_ nanoparticles. There is therefore a need for developing a PBPK model parameterized for CeO_2_ including deposition in the respiratory system and translocation/excretion.

The objectives of this study are:

1. Generate and characterize both fresh and UV-light aged CeO_2_ nanoparticles under general outdoor air composition.
2. Measure the biodistribution of CeO_2_ nanoparticles in rat following an inhalation study, including the main organs and feces and analyze the mass balance.
3. Develop, parameterize, and evaluate a PBPK model that combines pulmonary deposition with internal biodistribution to predict the biodistribution kinetic of inhaled CeO_2_ nanoparticles in rats.

## Methods

### Experimental apparatus

**Fig. 1:**
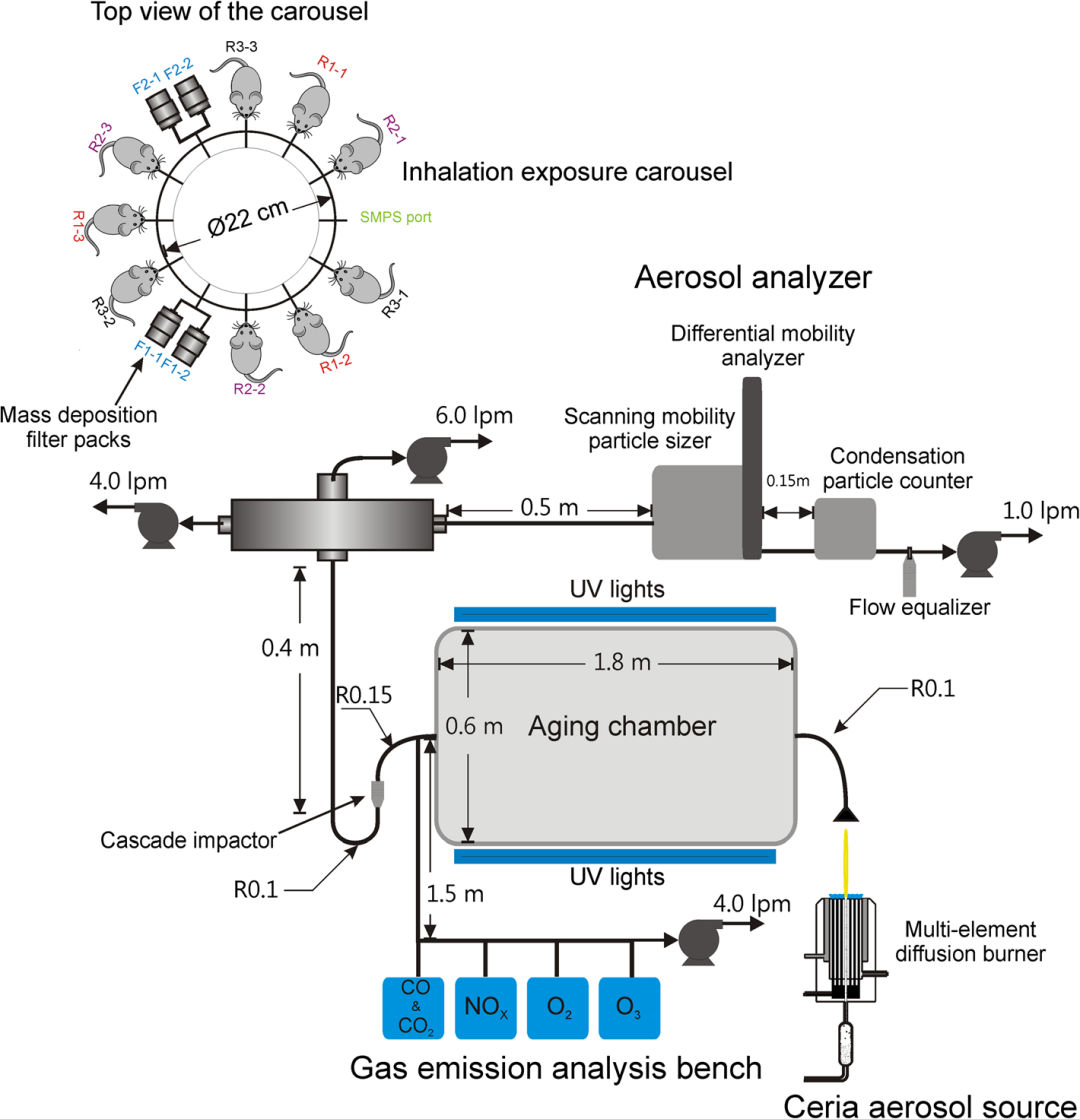
Schematic representation and photo of the experimental apparatus. The dimensions are not to scale. “lpm” stands for liters per minute. “R” notations beside tubing connections indicate radius and the associated numbers are in meters. The “F” and “R” notations beside the carousel represent filter pack and rat positions, respectively

Figure 1 shows the schematic of the experimental apparatus consisting of a nanoparticle generation facility, a photochemical aging chamber, a series of instruments to characterize the aerosol flow, and a nose-only inhalation exposure chamber for rats.

#### Generation of CeO_2_ nanoparticles

CeO_2_ nanoparticles were generated using the University of Michigan combustion synthesis (UMCS) facility which was transported to Michigan State University for the animal exposure studies. The UMCS facility uses a multi-element H_2_/O_2_ diffusion flame burner to provide a high-temperature oxidizing environment for cerium acetate (cerium (III) acetate hydrate; Sigma-Aldrich, 99.99 % trace metals basis) precursor decomposition, oxidation, and particle formation. The burner was operated continuously to generate appropriate quantities of nanoparticles for the exposure studies and analysis. Further information on the synthesis facility can be found in Bakrania et al. [28, 29], Miller et al. [30], Hall et al. [31], and (Fatouraie et al.: Combustion-generated particle synthesis and delivery system for inhalation exposure studies: case study for ceria nanoparticles, submitted).

#### Photochemical chamber

The atmospheric reaction chamber is a 500-L fluorinated ethylene propylene Teflon bag designed in collaboration with Ingeniven Inc. (North Hampton, NH, United States). Thirty (UVA-340) fluorescent lamps (40 W each) were used to simulate sunlight in the short wavelength region from 365 nm to the solar cutoff of 295 nm. The lamps were placed beside the chamber to simulate ~250 W/m^2^ of direct UV flux in the chamber, which is equivalent to five times the solar UV flux. Reflections from the interior surface of the enclosure are expected to significantly increase this figure and actual UV flux levels were measured with a UV meter. To simulate atmospheric aging conditions, the lamps were turned on simultaneously with the generation and exposure of CeO_2_ nanoparticles, with a high UV intensity of ~250 W/m^2^ of direct UV flux at a peak emission wavelength of 340 nm to accelerate aging and a residence time of the CeO_2_ nanoparticles of approximately 15 min in the aging chamber.

#### Nanoparticle characterization

A scanning mobility particle sizer (SMPS) with an electrostatic classifier (TSI-3080, TSI Inc., Shoreview, MN, USA) was used to monitor the aerosol number density and particle size distribution as a function of time during the animal exposure studies. Specifically, the size distribution of the aerosol was monitored during the experiments in real time using a differential mobility analyzer (DMA) (TSI-3081, TSI Inc., Shoreview, MN, USA) connected to a condensation particle counter (CPC) (TSI-3010, TSI Inc., Shoreview, MN, USA). The SMPS uses a particle size impactor filter to remove particles > ~0.5 μm in size. Additionally, a three-stage cascade impactor filter with cut-off stages at 2.5, 1, and 0.5 μm (Sioutas Impactor, SKC Inc., Eighty Four, PA, USA) was incorporated after the photochemical chamber, upstream of the exposure carousel, filter packs and SPMS, to remove larger particles and agglomerates > ~0.5 μm in size. The midpoint particle mobility diameter recorded by the SMPS was used to calculate the equivalent spherical volume of the aerosol, which was multiplied by the theoretical density of CeO_2_ (7.13 g/cm^3^) to estimate the average mass concentration. The mass concentration calculated from SMPS data was compared with the physical sampling measurements based on the filter packs.

Gas sampling (GS) was used to measure the NO_x_, CO, O_3_, and CO_2_ levels in the carrier gas of nanoparticles. The NO_x_ (NO and NO_2_ measured using a chemiluminescence analyzer, Thermo Scientific 42C, Thermo Fisher Scientific Inc) and O_3_ (measured using a UV photometric analyzer, Thermo Scientific 49C, Thermo Fisher Scientific Inc, USA) were by-products of the combustion process used to generate the ceria nanoparticles, and also represent gases found at ambient conditions in urban environments. Additional measurement of post combustion O_2_ levels were performed using an emission analyzer (MEXA-584 L, HORIBA Instruments, Ann Arbor, MI, USA) to ensure the oxygen concentration was at atmospheric levels, i.e. near 20 % on a mole fraction basis.

The mass of nanoparticles during each exposure study was measured using filter pack assemblies. Four 47-mm Teflon (PTFE) filters (Gelman Science) in Teflon/Teflon-coated aluminum filter packs were attached to two ports of the exposure chamber to determine the total nanoparticle mass deposited during sampling times corresponding to the exposure studies. The filter packs were used according to the Federal Reference Method [32]. Four pumps were used to control the flow rates to the exposure carousel, the filter packs, the SMPS and the GS system. Additional details on the aerosol transport system, including dimensions and flow rates, are described by (Fatouraie et al.: Combustion-generated particle synthesis and delivery system for inhalation exposure studies:case study for ceria nanoparticles, submitted).

Transmission electron microscopy (TEM) was performed at the University of Michigan electron microbeam analysis laboratory using a high resolution electron microscope (JEOL 3011, JEOL USA Inc., Peabody, MA, USA) in order to characterize the morphology and size distribution of the nanoparticles. The nanoparticles were sampled on copper grids (carbon film, 300 mesh copper, Electron Microscopy Sciences, Hatfield, PA, USA) placed in the aerosol flow after the impactor filter. Bulk properties such as crystalline structure and the average crystallite size were analyzed using powder x-ray diffraction (XRD) equipment (Bruker D8 Discover with GADDS, BRUKER AXS Inc., Madison, WI, USA). Details on the sample preparation for the nanoparticle materials analysis are described by (Fatouraie et al.: Combustion-generated particle synthesis and delivery system for inhalation exposure studies:case study for ceria nanoparticles, submitted).

### Animal study design

#### Animals and inhalation exposure

Male Sprague-Dawley rats weighting 200–235 g, 9 weeks of age, were obtained from Charles River Laboratories (Portage, MI, United States) and housed for at least 7 days in shoebox-style cages prior to experimental protocols with access to food and water *ad libitum*. Study protocols were approved by the Institutional Animal Care and Use Committee of Michigan State University, an AAALAC accredited institution.

Rats were acclimated to nose-only restraining devices (CH Technologies, Westwood, NJ, USA) for at least 4 days preceding exposure. For the experiments in this study, nine rats were exposed to CeO_2_ nanoparticles in a 12-port nose-only exposure system (CH Technologies) for a single 4-hour exposure. Two ports of the system were used to collect nanoparticle samples and one port was used to connect to the SMPS. The exposure environmental conditions were 25 ± 3 °C, 50 ± 15 % humidity, and the dilution levels in the burner were controlled to achieve oxygen concentration of 20 % on a mole fraction basis with upper limits of 25 ppb for O_3_ and 50 ppb for NOx. Two sets of experiments of nine rats per experiment were used for both freshly-generated CeO_2_ nanoparticles and aged CeO_2_ nanoparticles. After exposure, three rats each were sacrificed after 15 min, 24 h, and 7 days (*n* = 3 per time point per experiment). Blood, lungs, liver, kidneys, heart, brain, olfactory bulb, spleen, feces, and urine were collected for CeO_2_ nanoparticles concentration analysis. Feces and urine were collected only for the first 24 h post exposure. Gastrointestinal tract (GI tract) samples were also collected for analysis from the aged nanoparticles exposure experiments.

A separate pilot study was conducted to measure the evolution of CeO_2_ nanoparticle concentrations in the feces. Six rats were exposed to 770 ± 210 μg/m^3^ cerium oxide nanoparticles for 5 h and feces were collected 1, 2, 4, 5, and 7-day post exposure using metabolic cages. Exposure protocols are the same as described above.

#### Determination of CeO_2_ nanoparticle concentrations in biological samples

All equipment and supplies used for trace element analysis were rigorously acid-cleaned. Detailed procedures on the sample digestion and analysis were documented previously [33, 34]. In brief, all biological samples were weighed and then acid-digested in concentrated nitric acid. Sample extracts were then diluted and analyzed for cerium using inductively coupled plasma–mass spectrometry (ICP-MS) (ELEMENT2, Thermo Finnigan, San Jose, CA, USA). The detection limit of the method is 0.002 ng/mL. The background cerium levels in all sampled organs were determined in a preliminary study with two control rats which were not exposed to the CeO_2_ nanoparticle aerosol and did not undergo any sham exposure procedures. All background levels were below the detection limit.

The cerium concentration levels in all organs at all time points for all four sets of experiments were normalized by the corresponding exposure concentrations measured by the filter packs. The data was then grouped into three time points for fresh and aged nanoparticles with each time point having a larger sample size. Shapiro-Wilk test was performed to confirm the data was following normal distribution before A two-tailed heteroscedastic Student *t*-test was then performed to examine the null hypothesis of no significant difference between the biodistribution of fresh and aged nanoparticles for each time point and organ.

### PBPK modeling

#### Model framework

**Fig. 2:**
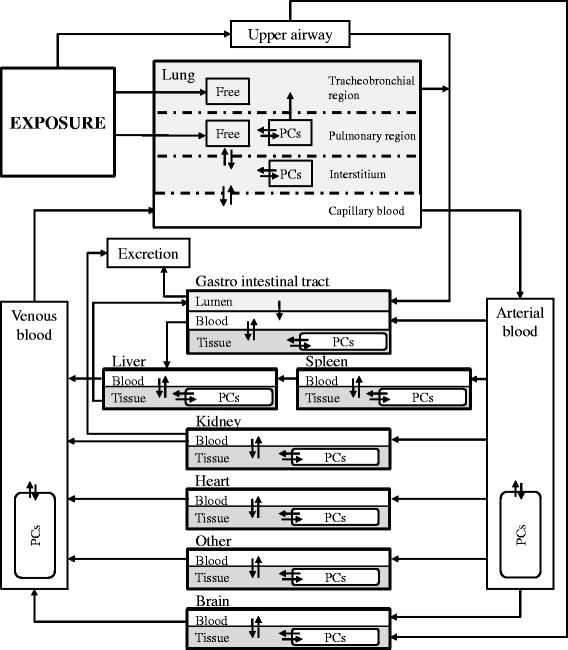
Schematic representation of the nanoparticle PBPK model developed in this study for inhalation, with sub-compartments of phagocytizing cells (PCs) in each tissue. *Arrows* represent the direction of various pathways for the nanoparticles to migrate from one location to another. Nanoparticles may not be endocytosed by PCs nor migrate into the tissue matrix and exist in a “free” state in the tracheobronchial and pulmonary region of the lung

This PBPK is based on our previous version for intravenously injected nanoparticles already published [23, 35], extended for inhalation exposure by including deposition in the respiratory system and transfer to the GI tract. The present PBPK model consists of ten compartments: arterial blood, venous blood, lungs, spleen, liver, kidneys, heart, brain, GI tract, and the rest of the body. All compartments are interconnected via systemic circulation (Fig. 2). Within each organ compartment, there are three sub-compartments representing capillary blood, tissue, and phagocytizing cells (PCs). The arterial blood and venous blood compartments also have PCs sub-compartments. In the experiments, nanoparticles were inhaled by the rats immediately at the start of exposure to the aerosol, and the model similarly started predicting uptake of particles immediately upon exposure. A similar structure used by Sweeney and co-workers was adopted to describe deposition and clearance in the respiratory system [27]. Following inhalation, the nanoparticles are deposited into three regions of the respiratory system: the upper airway, the tracheobronchial region, and the pulmonary region. The deposition fractions are fitted by observed amounts in different organs, with the same values used for all simulations of the four studies.

After being deposited in the upper airway, nanoparticles can migrate either to the brain via the olfactory bulb, or to the GI tract by being swallowed. Nanoparticles deposited in the tracheobronchial region can be transferred to the pharynx in the upper airway by mucociliary clearance, then swallowed to the GI tract lumen and eventually excreted via feces. After penetrating the interstitium of the lungs, nanoparticles can enter the systemic circulation system and migrate to other organs. PCs loaded with nanoparticles in the pulmonary region can also be transferred to the upper airway by mucociliary transportation.

The exchange of nanoparticles between blood and tissue in each organ is described as a flow- and diffusion-limited process. The flow-limited process is controlled by a permeability coefficient, which limits the effective blood flow [15]. We assume one permeability coefficient for the brain, a different permeability coefficient for the liver and the spleen, and another different permeability coefficient for the other organs [35]. The permeability coefficient for the brain compartment is expected to be much lower under the assumption of a highly efficient blood-brain barrier as observed by Hardas et al. [12]. The diffusion-limited process is controlled by the tissue:blood partition coefficient and assumed to be the same for all organ tissues [23].

A fraction of the nanoparticles entering the tissue are sequestrated by PCs until the PCs are eventually saturated. All PCs are assumed to behave the same but the abundance of PCs in the tissues differ. The effective uptake rate by PCs is a function of the maximum uptake rate and decreases as the PCs become saturated. The maximum uptake rate is the same for all compartments except for the spleen, due to its mesh-like structure that could trap nanoparticles in the spleen marginal zones and delay their contact with the splenic PCs [36, 37]. Nanoparticles can also re-enter the tissue after desorption from PCs by processes such as exocytosis [38, 39]. The uptake capacity and uptake rate for each PC may vary between different nanoparticles, while the relative abundance of PC densities in different organs was taken to be the same for all types of nanoparticle.

Excretion of nanoparticles occurs from the GI tract, liver tissue, and the capillary blood of the kidneys. Degradation of CeO_2_ nanoparticles is considered negligible for the time-scale of this study.

#### Main mathematical description of the model

We present here two main model equations describing the mass balances of a tissue *t* and of the PCs in this tissue. A more comprehensive derivation of the mathematical representation of the model is given in Additional file 1.

The dynamics of the nanoparticles in the tissue sub-compartment can be predominantly described by two mechanisms: 1) the transfer between arterial blood and venous blood and; 2) the interaction with the PCs. In certain organs, excretion of nanoparticles occurs as a clearance route. The equation describing these processes is:

1 $$ \frac{d{M}_t}{dt}=\overset{transfer\  between\  arterial/ venous\  blood}{\overbrace{\frac{\chi_{\alpha}\times {Q}_t}{\left(1+{\chi}_{\alpha}\right)}\times \left({C}_{art}-{C}_t/P\right)}}-\overset{interaction\  with\ PCs}{\overbrace{\left({W}_t\times {C}_t\times {k}_{t, ab}-{M}_{t,m}\times {k}_{de}\right)}}-\overset{clearance\ by\  excretion}{\overbrace{\frac{d{M}_{e, ex}}{dt}}} $$

where,

*M*_*t*_ [μg] – Amount of nanoparticles in the tissue of organ *t.*

*χ*_*α*_ [unitless] – Permeability coefficient between capillary blood and tissue.

*Q*_*t*_ [mL per hour] – Regional blood flow in organ *t*.

*C*_*art*_ [μg per g] – Concentration of nanoparticles in the arterial blood.

*C*_*t*_ [μg per g] – Concentration of nanoparticles in the tissue of organ *t*.

*P* [unitless] – Partition coefficient of nanoparticles between tissue and blood.

*W*_*t*_ [g] – Weight of organ *t*.

*M*_*t,m*_ [μg] – Amount of nanoparticles captured by the PCs in organ *t*.

*k*_*t,ab*_ [per hour] – Current uptake rate of nanoparticles by the PCs in organ *t.*

*k*_*de*_ [per hour] – Desorption rate of nanoparticles from the PCs to tissue.

*M*_*e,ex*_ [μg] – Amount to excreta from source *e. e* only applies to tissue in the liver and capillary blood in the kidneys. The elimination of nanoparticles directly from the GI tract is described separately (Additional file 1).

The change in mass of the nanoparticles in the PCs is the uptake from the tissue minus desorption from the PCs back to tissue. The uptake rate *k*_*t,ab*_ will decrease as the amount of nanoparticles captured approaches the total PCs saturation level, characterized by the PCs uptake capacity per unit weight. The equation describing these behaviors is:

2 $$ \frac{d{M}_{t,m}}{dt}=\overset{uptake\  from\  tissue}{\overbrace{W_t\times {C}_t\times k{}_{ab0}\times \left(1-\frac{M_{t,m}}{M_{t, cap}\times {W}_t}\right)}}-\overset{desorption\  back\ t\mathrm{o}\  tissue}{\overbrace{M_{t,m}\times {k}_{de}}} $$

where,

*k*_*ab0*_ [per hour] – Maximum uptake rate by the PCs (the same for all organs except the spleen [35]).

*M*_*t,cap*_ [μg per g] – PCs uptake capacity for nanoparticles per organ *t* weight, which is determined as the PCs number per organ weight (nanoparticle type independent) multiplied by the maximum uptake capacity in individual PCs (the same for all organs except the spleen) [35].

In addition, the model divides the respiratory system into three compartments; the pulmonary, tracheobronchial, and upper airway regions. In the pulmonary region, nanoparticles translocate into systemic circulation (described by first order kinetics) or become engulfed by PCs residing in the alveolar sacs (Eq. 2). The PCs in the pulmonary region transport nanoparticles to the tracheobronchial region for further clearance by a mucus escalator to the larynx and the GI tract, which is described by first order kinetics. In the upper airway region, the nanoparticles are cleared mainly to the GI tract, but also transferred to the olfactory system using first order transfer rates. More detailed information can be found in Additional file 1.

#### Model implementation

The PBPK model was implemented in Berkeley Madonna™ version 8.3.18 (Berkeley, CA) and acslX™ version 3.0.2.1 (Huntsville, AL). Parameters with unknown values were optimized by fitting the model parameters and comparing the model results with experimental data obtained from this study using the Nelder-Mead method in acslX™ with termination criteria set as 0.001, 0.001, and 1000 for parameter stop tolerance, object function stop, and maximum iteration. To create the most parsimonious model possible, common generic parameter values were used for most organs. Differentiated parameters between compartments were only used when it was indispensable to explain the dynamics of the CeO_2_ concentrations in that compartment in a physiologically meaningful way. The fraction of residual capillary blood left in the organs when analyzed for nanoparticle contents and the PCs uptake capacities in the organs were taken from our previous model, developed for intravenous injection [23] and tested on various nanoparticles [35]. All other parameter values were taken from the scientific literature [15, 40–43]. Values of all parameters are listed in Tables 1 and 2 with the newly fitted parameters in this study reported in bold with corresponding standard deviations. Since the study showed little difference between fresh and aged nanoparticles, neither for the CeO_2_ characterization, nor for their biodistribution, the same model parameters could be used for both fresh and aged nanoparticles.

**Table 1:** Description and values for non-inhalation specific pharmacokinetics parameters. Values in bold are fitted ^a^

Parameter (unit)	Description	Generic values ^b^	Spleen (s)	Liver (l)	Lungs (lu)	Heart (h)	Kidneys (k)	GI tract (gi)	Brain (br)	Rest of the body (rest)	Blood (blood)
*W*_*t*_ */WB (unitless)* ^b^	% of organ weight to body weight	-	0.0031	0.0396	0.0047	0.0034	0.0094	0.044	0.0053	0.84	0.0498
*W*_*t,b*_ */W*_*t*_ (unitless) ^c^	% of capillary blood in organ to organ weight	-	0.225	0.156	0.1	0.175	0.316	0.1	0.07	0.017	-
*frQ*_*t*_ (unitless) ^d^	Fraction of cardiac output to organ	-	0.0146	0.0465	1	0.051	0.141	0.213	0.02	0.514	1
*N*_*t*_ (# per g) ^d^	Phagocytizing cells number per organ weight	-	2.08 × 10^8^	2.72 × 10^7^	2.69 × 10^6^, **3.90 × 10** ^**6**^ **± 3.71 × 10** ^**2**^ ^g^	7.60 × 10^4^	9.90 × 10^4^	**5.06 × 10** ^**5**^ **± 2.22 × 10** ^**3**^	3.06 × 10^5^	8.11 × 10^6^	1.85 × 10^3^
*M*_*cap*_ (μg per #) ^f^	Maximum uptake capacity in individual phagocytizing cells	**5.52 × 10** ^**−7**^ **± 6.98 × 10** ^**−11**^	-	-	-	-	-	-	-	-	-
*χ*_α_ (unitless) ^f^	Permeability coefficient between blood and tissue	**7.76 × 10** ^**−1**^ **± 2.14 × 10** ^**−4**^	**Generic**	**Generic**	**Generic**	**Generic**	**Generic**	**Generic**	**6.75 × 10** ^**−7**^ **± 8.84 × 10** ^**−11**^	**1.71 × 10** ^**−2**^ **± 6.54 × 10** ^**−7**^	-
*P* (unitless) ^f^	Partition coefficient between tissue and blood	**2.09 × 10** ^**−1**^ **± 9.83 × 10** ^**−7**^	**Generic**	**Generic**	**Generic**	**Generic**	**Generic**	**Generic**	**Generic**	**Generic**	1
*k*_*ab0*_ (per h) ^f^	Maximum uptake rate by phagocytizing cells	**1.45 × 10** ^**0**^ **± 4.62 × 10** ^**−5**^	**5.18 × 10** ^**−1**^ **± 5.87 × 10** ^**−5**^	**Generic**	**Generic**	**Generic**	**Generic**	**Generic**	**Generic**	**Generic**	**Generic**
*k*_*de*_ (per h) ^f^	Desorption rate by phagocytizing cells	**5.30 × 10** ^**−19**^ **± 1.24 × 10** ^**−17**^	**Generic**	**Generic**	**Generic**	**Generic**	**Generic**	**Generic**	**Generic**	**Generic**	**Generic**
*CLE*_*e*_ (per h)	Clearance rate to excreta	-	-	**1.52 × 10** ^**−3**^ **± 6.97 × 10** ^**−6**^	-	-	**3.26 × 10** ^**0**^ **± 2.70 × 10** ^**−3**^	**1.41 × 10** ^**−1**^ **± 8.42 × 10** ^**−7**^	-	-	-
*fr*_*β*_ (unitless) ^e, f^	Fraction of capillary blood of organs left when analyzed	0.144	Generic	Generic	Generic	Generic	Generic	Generic	0.371	Generic	-

^a^ “-” stands for not applicable

^b^ Values were obtained by from Wenger et al. [45] except for the GI tract, which was obtained from Bernareggi and Rowland [42]

^c^ Values were obtained from literature estimates of the percentage (*w/w*) of capillary blood in the organs [41] except for lungs and GI tract, which were estimated by the authors

^d^ Values obtained from literature [15, 42, 43]

^e^ Values obtained from Carlander et al. [35]

^f^ *M*_*cap*_, *χα*, *P*, *k*_*ab0*_, *k*_*de,*_ *fr*_β_ have generic values for most compartments. “generic” indicates the corresponding generic value for each parameter

^g^ 2.69 × 10^6^ is for the lungs interstitium, 3.90 × 10^6^ ± 3.71 × 10^2^ is for the pulmonary region of the lungs

**Table 2:** Description and values for inhalation specific pharmacokinetics parameters All values are fitted

Parameter (unit)	Description	Value
*fr*_*ua*_ (unitless)	Fraction of inhaled nanoparticles deposited in the upper airway	2.11 × 10^−1^ ± 8.54 × 10^−7^
*fr*_*tra*_ (unitless)	Fraction of inhaled nanoparticles deposited in the tracheobronochial region	1.75 × 10^−2^ ± 1.23 × 10^−7^
*fr*_*pul*_ (unitless)	Fraction of inhaled nanoparticles deposited in the pulmonary region	4.36 × 10^−2^ ± 4.08 × 10^−8^
*k*_*giab*_ (per h)	Absorption rate of GI tract	5.41 × 10^−3^ ± 7.38 × 10^−8^
*k*_*uabr*_ (per h)	Transfer rate from upper airway to brain	8.36 × 10^−5^ ± 1.34 × 10^−9^
*k*_*uagi*_ (per h)	Transfer rate from upper airway to GI tract lumen	3.35 × 10^−1^ ± 2.24 × 10^−6^
*k*_*tragi*_ (per h)	Transfer rate from tracheobronchial region to GI tract lumen	5.52 × 10^−3^ ± 1.22 × 10^−6^
*k*_*pulmtra*_ (per h)	Transfer rate of inactive phagocytizing cells from pulmonary region to tracheobronchial region	8.65 × 10^−4^ ± 4.74 × 10^−7^
*k*_*lupi*_ (per h)	Transfer rate from pulmonary region to interstitium of lungs	1.26 × 10^−1^ ± 3.47 × 10^−5^
*k*_*luip*_ (per h)	Transfer rate from interstitium of lungs to pulmonary region	1.12 × 10^−6^ ± 2.49 × 10^−6^
*delay*_*gi*_ (h)	Time delay for nanoparticles to travel from respiratory system to GI tract	1.88 × 10^0^ ± 8.02 × 10^−5^
*delay*_*f*_ (h)	Time delay for nanoparticles in feces to be excreted out	7.9 × 10^0^ ± 1.04 × 10^−1^

#### Evaluation of the model and identification of key parameters

The same model evaluation approach was applied as in Li et al. [23]: we first visually inspected the output and then determined the deviation from the line of unity between the log_10_ of measured and predicted values [44], and calculated the corresponding R^2^ and squared geometric standard deviation from the unity line.

#### Sensitivity analysis

The sensitivities to each model parameter were measured by the relative change in area under the mass-time curve (AUC) divided by the relative change in each input parameter p taken at 1 %:

$$ \mathrm{Sensitivity}\ \mathrm{coefficient} = \frac{d\mathrm{A}\mathrm{U}\mathrm{C}/\mathrm{A}\mathrm{U}\mathrm{C}}{dp/p} $$

The sensitivity coefficients were calculated at 180 h after the start of exposure.

## Results

### Characterization of CeO_2_ nanoparticles

**Fig. 3:**
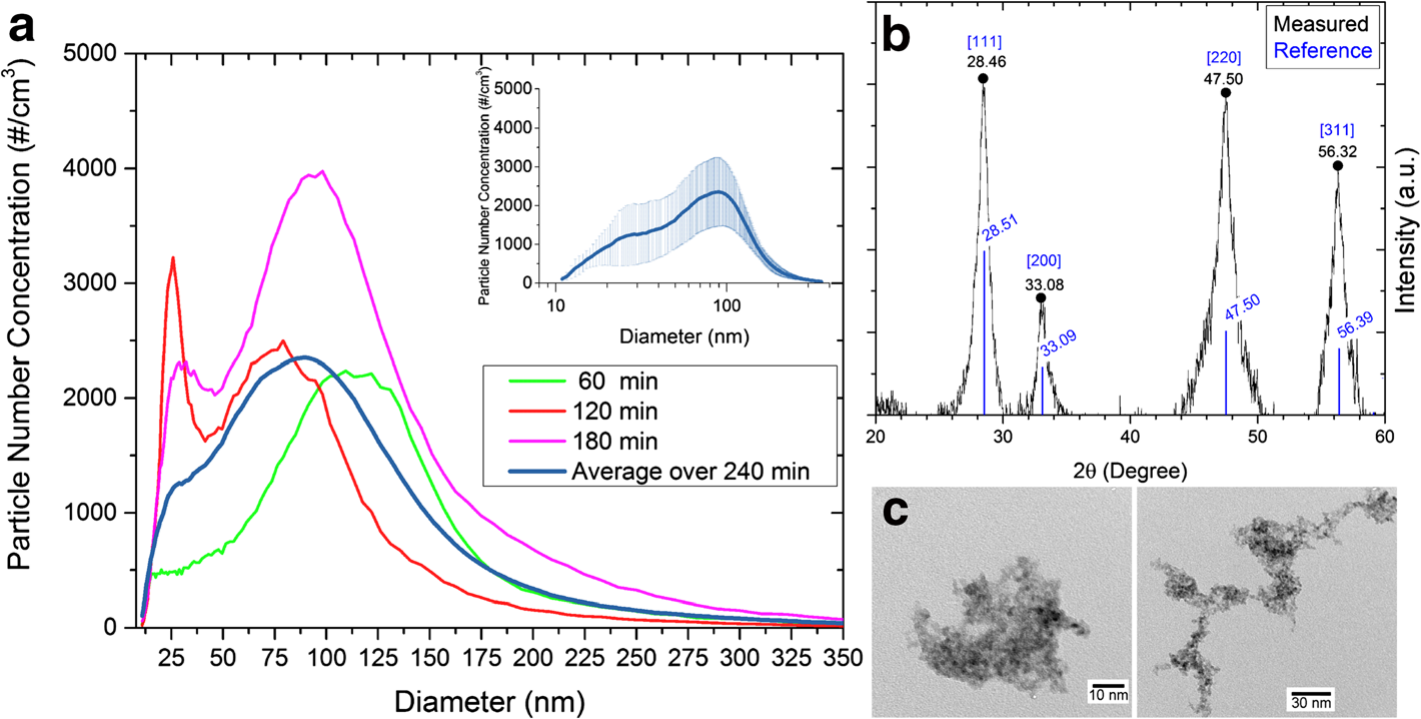
Characterization of the CeO_2_ nanoparticles. **a** SMPS results for number size distribution for CeO_2_ nanoparticles in the aged 2 experiment at 60, 120, and 180 min after the start of exposure. The average number size distribution for the 240-minute duration of the exposure study is presented in the panel and in the inset (log scale) where the error bars represent one standard deviation of the number size distribution for each particle size bin. **b** XRD spectra presented as a function of the XRD scattering angle 2θ of the powder sample collected on the impactor surface. The reference XRD spectra for CeO_2_ is presented for comparison. The labels correspond to the peak angles. **c** Bright field TEM images of CeO_2_ particles at two magnifications

A typical real time characterization of particle size distribution during an exposure study, as measured using the SMPS, is shown in Fig. 3a, for the second experiment of aged nanoparticles. The individual distributions are shown for 60, 120, and 180 min after the start of the experiments as well as the average particle size distribution over the 240 min of particle generation. The size distributions indicate slightly bimodal behavior with peaks around 25 and 90 nm, with ~90 % of the particles less than 200 nm in size. The estimates for the particle diameters are based on the equivalent diameter of a spherical particle with the same mass. The same size distribution trends were observed for both the fresh and aged particles. Additional details on the time history of the particle size distribution are provided by (Fatouraie et al.: Combustion-generated particle synthesis and delivery system for inhalation exposure studies:case study for ceria nanoparticles, submitted). After converting the number based concentrations to mass based concentrations, the size distribution for all four experiments follow a log-normal distribution (Additional file 2: Figure S1) with the following geometric means and 95 % confidence intervals: 146 nm (95 % confidence interval, 50 to 334 nm), 195 nm (95 % confidence interval, 66 to 334 nm), 174 nm (95 % confidence interval, 61 to 338 nm), and 151 nm (95 % confidence interval, 52 to 322 nm), for fresh 1, fresh 2, aged 1, and aged 2 experiments respectively.

The exposure concentrations based on filter pack measurements of CeO_2_ nanoparticles for experiments fresh 1, fresh 2, aged 1, and aged 2 were 172, 585, 483, and 439 μg/m^3^, respectively. During the fresh two experiment, exposure levels varied between an initial period of 197 min at a lower exposure level (when a leak at the exposure chamber was identified and repaired), and a subsequent period of 163 min with a higher exposure level, where 585 μg/m^3^ being the time-averaged concentration for the entire 6 h of exposure. The duration of the other three experiments was 4 h. The exposure concentrations based on the SMPS measurements were 361 μg/m^3^, 1240 μg/m^3^ (during the latter 163 min of the test), 619 μg/m^3^, and 444 μg/m^3^, for the fresh 1, fresh 2, aged 1, and aged 2 experiments, respectively. The SMPS based concentrations are relatively consistent with the concentrations based on the filter packs measurements except for the fresh 1 experiment. Based on the size distribution recorded by the SMPS, the exposure concentrations of nanoparticles smaller than 70 nm for the fresh 1, fresh 2, aged 1, and aged 2 experiments were 28.6 μg/m^3^, 39.8 μg/m^3^ (during the latter 163 min of the test), 27.0 μg/m^3^, and 36.5 μg/m^3^, respectively, showing less variation between the experiments than the total mass concentrations, which are biased to heavier/larger particles.

Bulk powder samples collected on the impactor surfaces were used for XRD analysis and to determine the phase, composition and average crystallite size of the nanoparticles. Because the nanoparticles for XRD analysis were taken from the impactor surface (to acquire sufficient material for analysis), the results are biased to larger particle sizes than the particles transported to the exposure chamber. However, the results are expected to be consistent for composition and phase data. Typical XRD spectra for fresh nanoparticles are presented in Fig. 3b. All peaks in the spectra correlate well with the crystallographic reference database for CeO_2_ [*Powder diffraction file, compiled by JCPDS. International Centre for Diffraction Data, Swarthmore, PA (1990)]*. The reference spectra are provided in Fig. 3b for comparison. Average crystallite size was determined based on the spectral peak broadening according to the Scherrer equation using the methods described in Bakrania et al. [29]. Detailed analysis was performed for two of the XRD features. An average crystallite size of 6.67 ± 0.06 nm was determined, which is as expected, slightly higher than the TEM values of primary particle diameter.

TEM images are presented in Fig. 3c for typical fresh nanoparticles. The images show the nanoparticles were highly agglomerated structures which consisted of small primary particles. TEM image analysis indicates the average diameter of the primary particles was typically 2–3 nm. Consistent with the SMPS data, analysis of the TEM images revealed no significant differences between the fresh and aged particles in terms of primary particle size or the agglomerated morphology.

Together the SMPS, XRD and TEM data indicate both fresh and aged nanoparticles are agglomerates of small primary particles of CeO_2_ approximately 2–3 nm in size, and the agglomerate dimensions (based on the SMPS data) span a size distribution up to ~200 nm in size.

### Biodistribution of CeO_2_ nanoparticles

Table 3 summarizes the concentrations of CeO_2_ nanoparticles found in the organs. The lungs consistently had the highest concentrations for all experiments, followed by the feces and the GI tract. The findings from the pilot study focused on feces showed (Additional file 3), compared to the first day post exposure, concentrations of CeO_2_ nanoparticles in the feces were reduced by 97 % on the fourth day post exposure and remained stable at low concentrations afterwards, as also observed by He et al. [13]. The evolution of the concentration in the GI tract also showed a decreasing trend (Table 3), although not as significant a rate of decay as observed for the feces. For the extrapulmonary organs (i.e., the blood, kidneys, heart, brain, liver, and spleen), different trends were observed. The concentrations in the blood showed a slight decrease over time. Concentrations in the spleen, liver, and kidneys rose steadily while concentrations in heart remained relatively stable. CeO_2_ nanoparticles were detected in the olfactory bulb and in the brain for the fresh 1 and fresh 2 experiments, while being mostly below the detection limit for the aged 1 experiment. The concentrations in the olfactory bulb were exceptionally high for the fresh 2 experiment (higher than in other extrapulmonary organs by one order of magnitude). For the aged 2 experiment, the brain had the highest concentrations among all experiments, while the concentrations in the olfactory bulb were below the detection limit except 1-day post exposure. The urine also had concentrations of CeO_2_ nanoparticles comparable to the blood concentration 1-day post exposure except for the fresh 2 experiment. The decreasing lung concentrations and the increasing concentrations in extrapulmonary organs suggests the CeO_2_ nanoparticles deposited in the lungs could serve as a secondary source of exposure to the extrapulmonary organs, either by direct transfer from the lungs or through an indirect route via the GI tract. Detailed concentrations for each rat are given in Additional file 4.

**Table 3:** Measured concentrations of CeO_2_ nanoparticles at different post exposure time (ng/g) ^a, b^

Organ	Fresh 1, filter packs based concentration 172 μg/m^3^, SMPS based <70 nm concentration 27.9 μg/m^3^			Fresh 2 ^c^, filter packs based concentration 585 μg/m^3^, SMPS based <70 nm concentration 38.9 μg/m^3^			Aged 1, filter packs based concentration 483 μg/m^3^, SMPS based <70 nm concentration 26.4 μg/m^3^			Aged 2, filter packs based concentration 439 μg/m^3^, SMPS based <70 nm concentration 35.6 μg/m^3^		
	15 min	1 day	7 day	15 min	1 day	7 day	15 min	1 day	7 day	15 min	1 day	7 day
Blood	3.10 ± 0.88	2.90 ± 0.22	1.85 ± 0.07	2.36 ± 0.33	2.32 ± 0.11	2.10 ± 0.35	1.50 ± 0.13	1.76 ± 0.24	0.91 ± 0.33	1.16 ± 0.29	0.57 ± 0.09	0.91 ± 0.35
Lungs	317 ± 129	225 ± 31	93.9 ± 129	1593 ± 144	935 ± 1317	1014 ± 1417	1307 ± 391	495 ± 469	775 ± 325	953 ± 758	1421 ± 250	929 ± 317
Spleen	0.92 ± 0.74	0.21 ± 0.07	2.26 ± 2.73	0.12 ± 0.05	0.46 ± 0.16	3.14 ± 4.39	0.34 ± 0.25	0.27 ± 0.02	0.38 ± 0.09	1.19 ± 0.07	1.55 ± 0.46	1.92 ± 0.24
Liver	0.49 ± 0.12	1.47 ± 0.22	6.65 ± 4.62	0.95 ± 0.22	1.55 ± 1.49	4.46 ± 5.63	0.31 ± 0.21	1.27 ± 0.99	6.36 ± 2.81	0.32 ± 0.15	1.63 ± 0.55	3.57 ± 0.64
Kidneys	0.22 ± 0.06	0.99 ± 0.35	1.67 ± 0.43	0.94 ± 0.03	1.41 ± 0.81	3.47 ± 3.21	0.24 ± 0.09	0.58 ± 0.11	1.73 ± 0.40	1.20 ± 0.16	1.93 ± 0.18	2.96 ± 0.54
GI tract	-	-	-	-	-	-	63.0 ± 30.4	1.26 ± 12.8	9.78 ± 4.95	34.4 ± 13.4	24.4 ± 20.4	8.95 ± 6.19
Heart	0.13 ± 0.03	0.37 ± 0.38	0.33 ± 0.06	0.61 ± 0.14	0.56 ± 0.13	0.66 ± 0.28	0.25 ± 0.19	0.49 ± 0.45	0.25 ± 0.07	1.36 ± 0.13	1.32 ± 0.15	1.47 ± 0.05
Brain	0.16 ± 0.01	5.55 ± 8.91	0.77 ± 0.11	1.11 ± 0.17	0.95 ± 0.03	1.42 ± 0.19	0.34 ^d^	BDL	BDL	1.64 ± 0.47	1.83 ± 0.76	1.41 ± 0.29
Olfactory bulb	0.69 ± 0.07	0.84 ± 0.08	0.69 ± 0.29	13.0 ± 10.6	5.92 ± 3.65	4.69 ± 1.71	BDL	BDL	BDL	BDL	0.92 ^d^	BDL
Feces	-	91.6 ± 4.3	-	-	769 ± 431	-	-	672 ± 431	-	-	656 ± 117	-
Urine	-	5.79 ± 3.37	-	-	-	-	-	1.66 ± 1.26	-	-	1.98 ± 2.06	-

^a^ All values displayed are mean ± standard deviation (*n* = 3)

^b^ “BDL” stands for below detection limit; “-” stands for no samples

^c^ The exposure duration for experiment fresh 2 was 6 h, instead of 4 h for all other experiments. Please refer to text for more details

^d^ Only one sample was above detection limit

The Shapiro-Wilk test showed the data was mostly following normal distribution. The Student *t*-test showed only the liver at 15-min post exposure, olfactory bulb at 1-day post exposure, and the blood for all three time points had *p*-value < 0.05. The null hypothesis of no significant differences between the biodistribution of fresh and aged nanoparticles for each time point and organ was therefore considered retained for all organs except for blood. For blood, the difference was due to the much higher normalized concentration in fresh 1 experiment, which could be the result of a higher sub-fraction of nanoparticles smaller than 70 nm found in that experiment (17 %, at least twice higher than the other experiments).

**Fig. 4:**
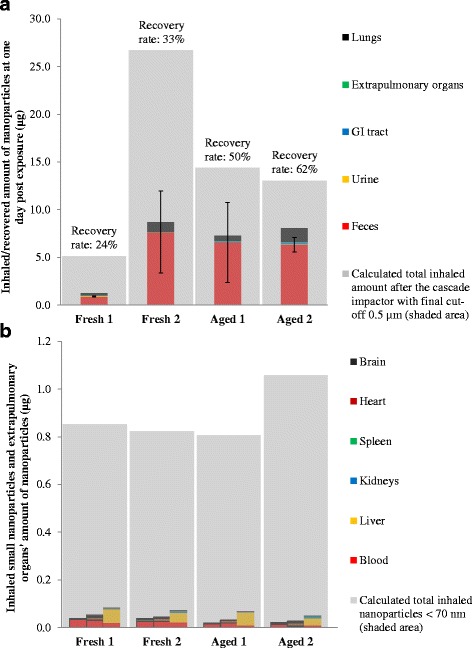
Biodistribution of CeO_2_ nanoparticles in different organs. **a** Mass balance of recovered amount 1-day post exposure compared with calculated total inhaled amount of nanoparticles based on the filter pack measurements after the cascade impactor (equipped with a final cut-off of 0.5 μm), error bars show one standard deviation of the feces data. **b** Evolution of the total nanoparticles mass in the extrapulmonary organs compared with the inhaled mass of nanoparticles less than 70 nm in size, identified using the SMPS measurements. The three bar stacks represent the data from 15-min, 24-h, and 7-day post exposure, respectively, from *left* to *right*

Combining the concentrations with the different organ weights, the masses of CeO_2_ nanoparticles were calculated and mass balance analysis was performed. The results are presented in Fig. 4, with Fig. 4a showing the total amount of nanoparticles recovered at 1-day post-exposure in comparison with the calculated total intake, and Fig. 4b showing the total amount of nanoparticles recovered in the extrapulmonary organs at 15 min, 1-day, and 7-day post-exposure in comparison with the sub-fraction of inhaled nanoparticles that were less than 70 nm as identified using the SMPS size distribution. The absolute recovered mass based on the ICP-MS measurements was approximately the same for all experiments but the fresh 1 experiment. The total estimated inhaled amount based on the filter pack measurements after the cascade impactor filter with the final stage cut-off of 0.5 μm was lowest for the fresh 1 experiment, the highest for the fresh 2 experiment, and was intermediary for the aged 1 and aged 2 experiments, with total inhaled mass estimates of 5.13, 26.69, 14.40, and 13.04 μg, respectively (see Fig. 4a). One-day post exposure, feces dominated the recovered masses (71–90 %) due to the high weight of the feces and the high concentrations of CeO_2_ nanoparticles (Fig. 4a). Although higher in concentration, the lungs had lower weights than the feces and therefore contributed less than the feces to the total recovered mass of CeO_2_, while remaining substantial (7–18 %). The urine and extrapulmonary organs both contributed between 4 and 6 % of the total recovered mass in the fresh 1 experiment, but contributed less than 0.5 % of the total recovered mass for the other experiments. Previous nanoparticle biodistribution studies have seldom included the GI tract in the analysis but the mass balance on the fresh nanoparticle exposure studies suggested that the GI tract could be accumulating a large proportion of the inhaled nanoparticles. To test this hypothesis, we analyzed the GI tract for the aged particle exposure studies. Nanoparticles found in the GI tract for the aged experiments contributed to 2 to 3 % of the total recovered mass, which is an order of magnitude higher than the total amount recovered in all extrapulmonary organs for the same experiments at 1-day post exposure. The total inhaled mass of nanoparticles smaller than 70 nm, based on the SMPS measured concentrations for the fresh 1, fresh 2 (corrected for the leakage), aged 1, and aged 2 experiments were 0.85, 0.82, 0.81, and 1.06 μg, respectively (Fig. 4b). Interestingly, the absolute amounts of nanoparticles found in the extrapulmonary organs varied by less than 65 %, while the total estimated inhaled amount based on filter pack measurements varied by a factor of five. The results may suggest only the fraction of CeO_2_ nanoparticles that are small enough can penetrate the alveolar wall and find their way into the extrapulmonary organs. The muscle, skin, fur, and skeletons of the rats were not analyzed for CeO_2_ contents. The unrecorded mass of nanoparticles in these tissues could explain the lower than 100 % recovery for the experiments.

### PBPK model simulation

**Fig. 5:**
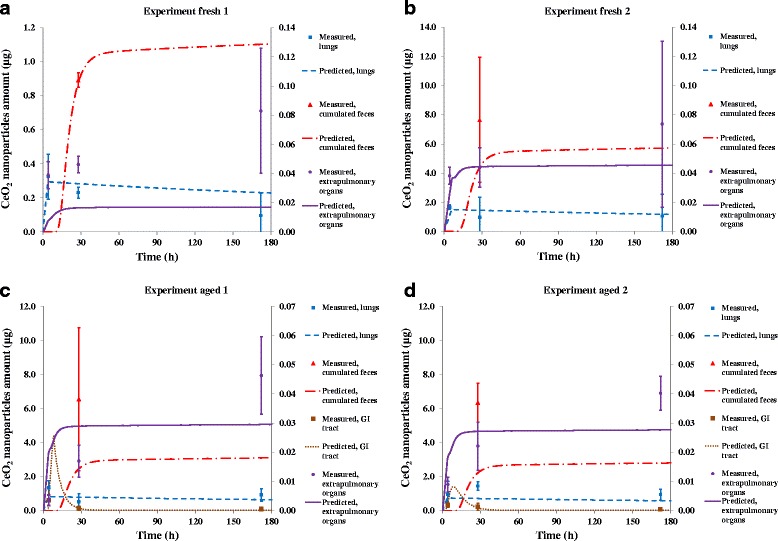
Predicted amounts of CeO_2_ nanoparticles compared with measured amounts in different organs. Extrapulmonary organs displayed on the secondary (right) y-axis. Error bars represent one standard deviation. Sample size for each measured data point and corresponding standard deviation shown was three rats. Predicted amounts were output from the PBPK model after optimization with the measured data obtained in this study. **a** Results for experiment fresh 1. **b** Results for experiment fresh 2. **c** Results for experiment aged 1. **d** Results for experiment aged 2

Figure 5 shows the evolution of the amount of nanoparticles predicted by the PBPK model and compares the results with the observed experimental data. The model results are able to reproduce the different experimentally observed trends. The CeO_2_ nanoparticle levels in the lungs increase rapidly during the exposure period, peak at the end of exposure, and then slowly decrease over time. After a period of delay, cumulated CeO_2_ nanoparticles amounts in the excreted feces increase rapidly until around the third day after exposure, before entering a phase of much slower increase [45]. The total amount of CeO_2_ nanoparticles in the extrapulmonary organs is much smaller than the amount in the feces and lungs. This amount increases quickly during the exposure period like what is seen in the lungs, but the amount keeps increasing after exposure until the end of the experiment for the spleen, liver, and kidneys (Fig. 5b and Additional file 5: Figure S3) in contrast to the decreasing levels in the lungs. The amount of CeO_2_ nanoparticles in the GI tract reach a maximum shortly after exposure and then decrease sharply, corresponding to the same time a large increase of CeO_2_ nanoparticles is observed in the feces (Fig. 5c and d). The model underestimates the amount of CeO_2_ nanoparticles in the feces and overestimates the decrease rate of CeO_2_ nanoparticles in the GI tract after exposure.

When examining individual organs (Additional file 5), the model suggests the CeO_2_ nanoparticles are almost exclusively (over 99 %) stored in the PCs in all organs except for the blood for the entire time span of the study. This indicates according to the model, the PCs in the organs are not yet saturated. For the blood, the transfer of CeO_2_ nanoparticles from the alveolar region to the blood during the exposure period overwhelms the uptake by the PCs in the blood. After exposure ends, there is still a considerable amount of CeO_2_ nanoparticles not captured by the PCs in the blood, which is because the PCs in the blood are saturated due to their low number. These “free” CeO_2_ nanoparticles in the blood then circulate to the extrapulmonary organs and are captured by the PCs in those organs over time.

**Fig. 6:**
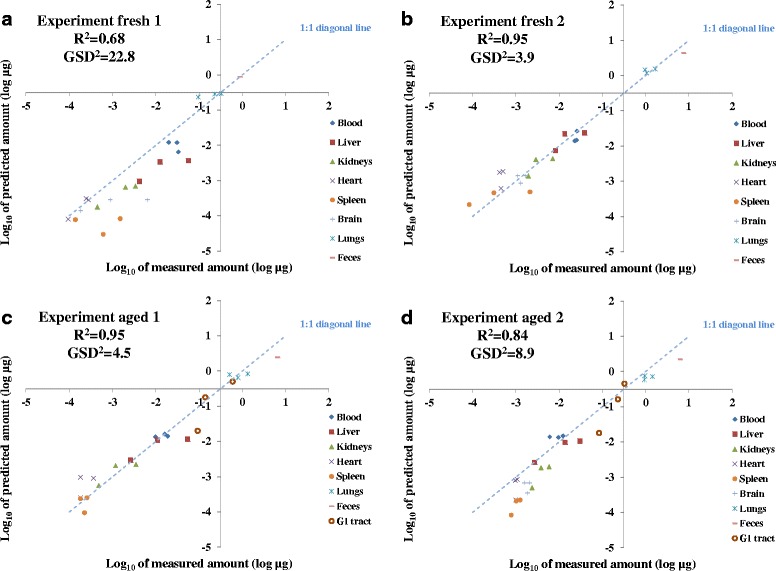
Log_10_ of predicted CeO_2_ nanoparticle amount as a function of log_10_ of measured amount in different organs. R^2^ represents the deviation from the line of unity between the log_10_ of measured and PBPK model predicted values. With *R* ^2^ = 1 representing perfect prediction of the measured CeO_2_ nanoparticle amount from the PBPK model. GSD^2^ indicates the differences (in factors) between the predicted and measured amounts of individual data points for all organs at all times. **a** Results for experiment fresh 1. **b** Results for experiment fresh 2. **c** Results for experiment aged 1. **d** Results for experiment aged 2

As illustrated in Figs. 5 and 6, the model predictions for the four experiments in this study agree generally well with measured data (R^2^ on the log scale ranging from 0.68 to 0.95). The GSD^2^ ranged from 3.9 to 22.8, indicating the accuracies of the predictions of individual data points are between a factor of 4 to 23 compared with the variation of four orders of magnitude differences between the CeO_2_ loadings in all organs at all times.

Table 4 summarizes the sensitivity of all parameters that are both nanoparticle-specific and show a sensitivity coefficient higher than 0.1 or lower than −0.1 in more than one organ. The parameters that show high sensitivity (higher than 0.3 or lower than −0.3) in almost all organs are the fraction of inhaled nanoparticles deposited in the upper airway (*fr*_*ua*_), and the fraction of inhaled nanoparticles in the pulmonary region (*fr*_*pul*_). The feces clearance rate from the GI tract (*CLE*_*fgi*_), the absorption rate of the GI tract (*k*_*giab*_), the partition coefficient between the tissue and the blood (*P*), and the permeability coefficient between the blood and tissues other than the liver and the spleen (*χ*_*rest*_) exhibit high sensitivity for most extrapulmonary organs. The blood is very sensitive to the maximum uptake capacity in individual PCs (*M*_*cap*_), but for all other organs this is not a very sensitive parameter. The brain is very sensitive to the transfer rate from the upper airway region to the GI tract (*k*_*uagi*_) while the other organs are not sensitive. The maximum uptake rate by the PCs (*k*_*ab0*_) is particularly sensitive for the spleen and urine.

**Table 4:** Sensitivity coefficients of important nanoparticle-specific parameters (four experiments averages, <−0.3 or >0.3 bolded)

Parameter	Blood	Lungs	Liver	Spleen	Kidneys	Heart	Brain	GI tract	Urine	Feces
*CLE*_*fgi*_^*a*^	−0.08	0.00	**−0.65**	**−0.62**	**−0.64**	**−0.46**	−0.17	**−0.92**	**−0.66**	0.08
*M*_*cap*_	**0.86**	0.01	−0.13	−0.09	−0.12	−0.07	−0.02	−0.01	−0.16	0.00
*k*_*ab0*_	−0.02	0.07	0.19	**−0.75**	0.14	0.10	0.02	0.00	**−0.81**	0.00
*k*_*giab*_	0.08	0.00	**0.69**	**0.66**	**0.67**	**0.49**	0.18	0.03	**0.70**	−0.04
*k*_*uagi*_	0.00	0.00	0.01	0.01	0.01	−0.23	**−0.72**	0.00	0.01	0.02
*P*	−0.05	0.00	**0.52**	**0.49**	**0.48**	**0.34**	0.12	0.04	**−0.46**	0.00
*χ*_*rest*_^*b*^	−0.05	0.00	**−0.39**	**−0.39**	**−0.36**	−0.27	−0.08	−0.02	**−0.41**	0.00
*fr*_*ua*_	0.08	0.00	**0.69**	**0.66**	**0.68**	**0.67**	**0.98**	**0.92**	**0.70**	**0.97**
*fr*_*pul*_	0.06	**0.76**	**0.42**	**0.42**	**0.43**	**0.42**	0.00	0.05	**0.44**	0.00

^a^ Clearance rate from GI tract to feces

^b^ Permeability coefficient between blood to rest of the body

## Discussion

The experimental design of this study enabled comparison of freshly generated and UV-light aged CeO_2_ nanoparticles with other conditions that may affect the characteristics of the nanoparticles held constant. As the other conditions were well controlled, any differences in the characteristics of the fresh and aged CeO_2_ nanoparticles are solely explained by the UV-light aging process. However, based on the results obtained from this study, the size distribution, morphology, and crystalline structure of the fresh and aged CeO_2_ nanoparticles were similar. Other studies showed under irradiation, some nanoparticles may have altered surface chemistry that can result in different toxicity or behaviors in the environment [46, 47]. Within the uncertainty of the current measurements, such effects were not apparent in the results for materials characterization or the biodistribution of the CeO_2_ nanoparticles. Further experiments using nanoparticles with controlled changes in surface chemistry would be of considerable interest to isolate the effects of surface chemistry. Also, considering CeO_2_ nanoparticles are used as additives to diesel fuel, another aging condition relevant for CeO_2_ would be to age CeO_2_ nanoparticles with soot co-produced from the combustion system. Such experiments could provide additional insights on the aging process under conditions different from the ambient urban environment and UV exposure represented in this study. For example, previous studies have found when CeO_2_ nanoparticles were produced with other co-pollutants from diesel engines, the CeO_2_ nanoparticles tended to agglomerate with soot particles instead of being freely dispersed [48, 49]. In addition, other reactive atmospheric compounds such as isoprene may also affect the result of the CeO_2_ aging process [50]. The results of the characterization of the CeO_2_ nanoparticles in this study indicated the effects of aging on morphology, size distribution and crystalline phase were minimal when CeO_2_ nanoparticles were subjected to a representative ambient urban environment, but not co-produced with soot. This suggests that soot is a critical component to the aging of CeO_2_ nanoparticles observed in previous studies.

Performing mass balance analysis is critical to ensure the majority of the CeO_2_ nanoparticles inhaled were accounted for in the biodistribution. More than half of the recovered nanoparticles were found in the feces. Data on the feces showed substantial variation that may be caused by a number of factors such as the amount of nanoparticles deposited on rats’ snout, whiskers and fur, the possible subsequent grooming by the rats that may lead to ingestion of externally-deposited nanoparticles during and post exposure, and contamination of the collected sample feces by rat fur. The high concentrations in the urine samples could actually be the result of contamination by the feces as urine and feces were in contact in the metabolic cages before being analyzed. Additionally, increased agglomeration of the nanoparticles may result in increased deposition in the upper airway region and if swallowed can lead to increased levels in the feces.

According to the filter packs measurements, experiment fresh 1 had a lower concentration of CeO_2_ nanoparticles. This was due to the lower count of larger nanoparticles in that particular experiment. The different size distributions of the nanoparticles in this experiment provided the opportunity to study the possible effects of particle size on uptake and biodistribution. Despite the differences in total inhaled dose among the four experiments (up to a factor of five difference), the amount of CeO_2_ recovered in all the extrapulmonary organs was quite similar (within 65 % of each other). Additionally, the amount of smaller CeO_2_ nanoparticles, with sizes <70 nm based on the SMPS data, varied by less than 31 %. Translocation across the alveolar epithelial monolayer has been demonstrated in in vitro experiments even for nanoparticles of primary particle size one order of magnitude larger than those in this study [51]. The higher degree of similarity between the amount of CeO_2_ recovered in the extrapulmonary organs and the amount of smaller sized nanoparticles could indicate only the smaller nanoparticles enter from the lungs and then travel to the extrapulmonary organs via blood circulation. Previous studies have confirmed this hypothesis [52, 53]. In addition, the overall translocated fractions, defined as the total amount of CeO_2_ in the extrapulmonary organs divided by the average amount in the lungs, increased from 4.5 to 10 % between 15 min and 7 days after exposure. This agrees well with a recent finding on the kinetics of nanoparticle translocation across the lung epithelial tissue barrier [26].

In this study, although the concentrations of CeO_2_ nanoparticles found in the brain and olfactory bulb were not consistently above the detection limit, the detected concentrations in the brain were of the same order of magnitude as found in the blood. This could support the hypothesis proposed by others that nanoparticles can enter the brain through the olfactory bulb [54, 55].

Besides the feces, most of the recovered CeO_2_ nanoparticles were found in the lungs for all four experiments in this study, which is consistent with previous studies [8, 13, 14]. In contrast to He et al. [13], this study showed higher amounts recovered in the feces than in the lungs. The difference may be due to differences in the exposure methods. He et al. used intratracheal instillation and a bolus dose, which will limit nanoparticle deposition outside the respiratory system and exclude the possibility of ingestion intake to the GI tract from the nanoparticles deposited externally on the snout and whiskers. In this study, the concentrations of nanoparticles found in the GI tract were at least an order of magnitude higher than data reported by He et al. Among the extrapulmonary organs, the liver and the blood had the highest amount of CeO_2_ nanoparticles. The amount of nanoparticles in the liver, spleen, and kidney increased over time; the amount in the blood decreased over time; and the amount in the heart and brain stayed relatively constant. These trends generally agree with previous pulmonary studies [13, 14] although the blood concentration reported by He et al. [13] kept increasing and the spleen results from Geraets et al. [14] did not show any clear trend as a function of time. Aalapati et al. [8] exposed mice to CeO_2_ nanoparticles daily for 4 weeks and examined the contents in different organs 2 and 4 weeks after the last exposure. They found a steady decrease in nanoparticle concentrations in both the lungs and the extrapulmonary organs. CeO_2_ nanoparticles might have been cleared on a longer time frame. However, this decrease was not found by He et al. [13] after 4 weeks of instillation in their study nor in the current work in the 1-week post exposure measurements. Nevertheless, the nanoparticles in the above mentioned studies are not exactly the same in terms of size and other characteristics and this difference could contribute to the varying results observed by different researchers.

Using diameters of the nanoparticles obtained from the SMPS measurements, our fitted values for the deposition fractions in the tracheobronchial and pulmonary regions are lower than that calculated from the Multiple-Path Particle Dosimetry Model (MPPD v2.11) while the deposition fraction in the upper airway region is higher (Additional file 2). This can be explained by the likelihood of agglomeration of nanoparticles when inhaled. The size of the agglomerate could increase and therefore increase deposition in the upper airway, and decrease deposition in the tracheobronchial and pulmonary regions.

The PBPK model in this study accounts for multiple physiological mechanisms to predict and explain the dynamics of the CeO_2_ nanoparticle levels in different organs. In contrast to Bachler et al. [25], the PBPK model developed in this work indicates CeO_2_ nanoparticle excretion in the feces predominantly originates from the GI tract lumen as a result of mucociliary clearance, not biliary excretion from the liver. This pathway is also supported by the high concentrations of nanoparticles found in the GI tract samples, which were generally one or two orders of magnitude higher than the concentrations found in the liver. The sources of nanoparticles in the GI tract lumen are a mixture of biliary excretion from the liver, and the transfer of either free nanoparticles or saturated PCs from the upper airway and pulmonary region by mucociliary movement to the larynx where the nanoparticles or saturated PCs are then swallowed to the GI tract. It is suspected the mucociliary clearance of nanoparticle-loaded PCs is responsible for the continuous excretion throughout the study. This transport of nanoparticle-saturated PCs from the lungs to the GI tract then to the feces is also the major contributor of the slow decrease of CeO_2_ nanoparticles out of the lungs and a widely accepted clearance route for inhaled nanoparticles [53, 56, 57]. Due to limited data as a function of time, the dynamics of the nanoparticles in the GI tract predicted by the model which included a rapid increase shortly after exposure followed by a rapid decrease cannot be verified. It would be of interest to have finer time resolution data on the short term after inhalation exposure for the GI tract data to confirm if the model predictions are true. The underestimate of the amounts of CeO_2_ nanoparticles in the feces and overestimate of the decrease rate of CeO_2_ nanoparticles in the GI tract after exposure by the model could be explained by not having a route to simulate the possible ingestion of nanoparticles after inhalation exposure, when the animals might have licked their furs that were deposited with nanoparticles. The PBPK model predicts the majority of the nanoparticles in the lungs are captured by the PCs in the pulmonary region. This agrees well with microscopic evidence from both intratracheal instillation of silver nanoparticles [53] and whole body inhalation exposure of CeO_2_ nanoparticles [58].

According to the PBPK model, the majority of the nanoparticles entering the blood are coming from the uptake of the nanoparticles in the pulmonary region during the exposure period. After exposure, some of the nanoparticles transported to the GI tract may be absorbed before being excreted during the fecal retention time. The absorbed nanoparticles from the GI tract, together with small amounts of nanoparticles that are desorbed from the PCs in the pulmonary region, enter the systemic circulation and increase the levels of nanoparticles in the extrapulmonary organs or keep the nanoparticle concentrations constant throughout the study. The phagocytosis of nanoparticles is a well-documented phenomenon [20, 21, 23] and supports the model predictions that most of the CeO_2_ nanoparticles are captured by PCs in the different organs. It is noteworthy that the level of exposure in this study is much lower than other CeO_2_ nanoparticle pulmonary exposure studies [8, 13, 14]. The concentrations in the extrapulmonary organs were also much lower than for studies with intravenously injected nanoparticles [10, 11, 45]. Thus, the PCs in all organs including the pulmonary region in the lungs are not saturated in the current model, leading to a limited amount of free nanoparticle that can translocate in the organs.

Compared with the intravenous nanoparticle PBPK model [23], this PBPK model is also sensitive to the partition coefficients between the tissue and the blood, the permeability coefficient between the blood and the tissue, and the maximum uptake rate of the PCs. This model however, is not sensitive to the PCs uptake capacity except for the blood compartment. This is because the blood level of nanoparticles in this inhalation study is far lower than the level in the intravenous injection experiment the previous model built upon. Therefore, the PCs in this study are far from being saturated, making the model insensitive to the PCs uptake capacity. As shown in the results, the model is sensitive to the clearance rate to the feces from the GI tract and the absorption rate of the GI tract. This implies the importance of the GI tract in the biodistribution of inhaled nanoparticles. The fractions of inhaled nanoparticles to the upper airway and pulmonary region are directly related to the size of the nanoparticles and are both among the most sensitive parameters of this model. This supports the expected conclusion that size is one of the most important properties of nanoparticles in regards to their biodistribution via inhalation. In contrast, the model is far less sensitive to the deposition fraction of inhaled nanoparticles to the tracheobronchial region. This is because the tracheobronchial region is neither an important point of entry nor a significant point of exit for the nanoparticles and only acts as a transitional node between the upper airways, the pulmonary region, and the GI tract.

Most PBPK models for nanoparticles are only applicable to intravenous injection [16–19, 23]. Our model is one of the few existing PBPK models that can predict the biodistribution of inhaled nanoparticles [25–27], and advances the field by taking into account the dynamic of nanoparticles in the feces directly excreted from mucociliary clearance from the lungs, which has been shown to be a major pathway for nanoparticles in this study and in the study by He et al. [13]. Nevertheless, our model also has some limitations when compared with the experimental results of this study. First, the amount of CeO_2_ nanoparticles in the liver is systematically underestimated at the end of this study. Our model could not predict an increase over time large enough to reach the measured levels 7-day post exposure (Additional file 5). Certain physiologic pathways may have been disregarded that cause this increase in the liver. For example, the nanoparticles may undergo endocytosis by the M cells in the GI tract for a period of time before being exocytosed and may be in contact with the GI tract wall [59]. This may create a delayed effect for the nanoparticles to be absorbed by the GI tract and make the nanoparticles in the GI tract a source that can slowly release free nanoparticles over time. Not including lymph nodes in the model may also play a role in the limitation of this PBPK model, as nanoparticles taken up by the lymph nodes can theoretically have delayed entry to the blood system via vena cava [60, 61].

Another limitation of the study is the large variation in some of the experimental data. The PBPK model is able to predict individual data points within one order of magnitude. However, some of the measured data displayed large variations identified by large standard deviations (see Table 3). The variation in the measured data could be caused by the individual behavior of the rats during and post exposure which can result in different intake of CeO_2_ nanoparticles.

## Conclusions

CeO_2_ nanoparticles were generated before being inhaled by rats in one integrated system in this study. Characterizations of fresh and aged nanoparticles, where the aged nanoparticles were exposed to UV light and urban air environments before inhalation, showed little differences in size distribution, morphology, or crystalline structure. The biodistribution of fresh and aged CeO_2_ nanoparticles followed the same patterns, with the highest amounts recovered in the feces and the lungs. The slow decrease of nanoparticle concentration in the lungs is explained by clearance to the GI tract and then to the feces. For the extrapulmonary organs, the level of nanoparticles in the blood reached a maximum at the end of exposure and then decreased. The PBPK model successfully predicted the dynamic of the CeO_2_ nanoparticles in the various organs measured in this study. The model suggests most of the nanoparticles were captured by PCs, which agrees with the literature. When applying this model to other nanoparticle inhalation studies, the exposure conditions including the size distribution of the nanoparticles should be clearly defined as these would affect model predictions.

## Additional files

Additional file 1: Mathematical representation of the model. (DOCX 79 kb)

Additional file 2: Calculation of the deposition fractions in different regions of the respiratory system. **Figure S1.** Size distribution of the mass based concentration of CeO_2_ nanoparticles for four experiments based on the SMPS collected data. (DOCX 57 kb)

Additional file 3: Figure S2. Concentrations found in feces in the pilot study. Error bars representing one standard deviation on the means. (DOCX 37 kb)

Additional file 4: Individual organ concentrations of CeO_2_ nanoparticles for all experiments. (DOCX 39 kb)

Additional file 5: Figure S3. Time history of nanoparticles in the individual organs for all experiments. (DOCX 122 kb)

## Abbreviations

CeO_2_: Cerium Oxide
GI tract: Gastrointestinal Tract
ICP-MS: Inductively Coupled Plasma–Mass Spectrometry
PBPK: Physiologically Based Pharmacokinetic
SMPS: Scanning Mobility Particle Sizer
