# Supplementary material for: In vivo biodistribution and physiologically based pharmacokinetic modeling of inhaled fresh and aged cerium oxide nanoparticles in rats
Source: Part Fibre Toxicol. 2016 Aug 20;13:45. doi: 10.1186/s12989-016-0156-2 (PMC4992249; doi:10.1186/s12989-016-0156-2)
Supplement: Supplementary file 1 — Mathematical representation of the model. (DOCX 79 kb) [file 12989_2016_156_MOESM1_ESM.docx]

**Additional file 2 for**

*In vivo* biodistribution and physiologically based pharmacokinetic modeling of inhaled fresh and aged cerium oxide nanoparticles in rats

Mathematical representation of the model

S2.1 Calculation of total inhaled nanoparticles

The total amount of nanoparticles inhaled, expressed in µg, during the study is described by the following equation:

$\text{intake}=Conc\times ExpedT\times BF\times TV$ S1

where

*intake* [µg] – Total inhaled amount of nanoparticles during the study.

*Conc* [µg/m^3^] – Concentration of nanoparticles in the air.

*ExpedT* [hour] – Exposed duration.

*BF* [per hour] – Breathing frequency.

*TV* [m^3^] – Tidal volume.

The values for *Conc* and *ExpedT* follow the study settings. The values for *BF* and *TV* are deducted from the body weights of rats based on Stahl’s study [1]. The *ExpedT* is a dynamic parameters with a value starting from 0 to the total number of hours of the exposure duration.

*Inflows and outflows in different regions of the respiratory system and GI tract lumen*

After being inhaled, the nanoparticles deposit in different regions of the respiratory system. They can then exchange with nearby organs. The GI tract lumen is relevant here because nanoparticles in the upper airway and tracheobronchial region can be swallowed. The inflows and outflows for the different regions of the respiratory system and GI tract lumen are described as below.

Pulmonary region

$\frac{dM_{pul}}{dt}=\frac{\text{intake}}{ExpedT}\times fr_{pul}+M_{lu}\times k_{luip}-M_{pul}\times k_{\text{lupi}}-\frac{dM_{pul,m}}{dt}$ S2

where

*M_pul_* [µg] – Amount of nanoparticles in the pulmonary region.

*fr_pul_* [unitless] – Fraction of nanoparticles deposited in the pulmonary region.

*M_lu_* [µg] – Amount of nanoparticles in the rest of the lung.

*k_luip_* [per hour] – Transfer rate from interstitium of lungs to pulmonary region.

*k_lupi_* [per hour] – Transfer rate from pulmonary region to interstitium of lungs.

The last term describes the interaction with phagocytizing cells (PCs), which is described in details in the following sections.

Tracheobronchial region

$\frac{dM_{tra}}{dt}=\frac{\text{intake}}{ExpedT}\times fr_{tra}+M_{pulm}\times k_{pulmtra}-M_{tra}\times k_{tragi}$ S3

where

*M_tra_* [µg] – Amount of nanoparticles in the tracheobronchial region.

*fr_tra_* [unitless] –Fraction of nanoparticles deposited in the tracheobronchial region.

*M_pulm_* [µg] – Amount of nanoparticles in the pulmonary region PCs.

*k_pulmtra_* [per hour] – Transfer rate of saturated PCs from the pulmonary region to the tracheobronchial region.

*k_tragi_* [per hour] – Transfer rate of nanoparticles from the tracheobronchial region to the GI tract.

Upper airway region

$\frac{dM_{\text{ua}}}{dt}=\frac{\text{intake}}{ExpedT}\times fr_{ua}-M_{ua}\times\text{（}k_{uabr}+k_{uagi})$ S4

where

*M_ua_* [µg] – Amount of nanoparticles in the upper airway region.

*fr_ua_* [unitless] –Fraction of nanoparticles deposited in the upper airway region.

*k_uabr_* [per hour] – Transfer rate of nanoparticles from the upper airway region to the brain.

*k_uagi_* [per hour] – Transfer rate of nanoparticles from the upper airway to the GI tract.

GI tract lumen

$\frac{dM_{gilumen}}{dt}=M_{tra}\times k_{tragi}+M_{ua}\times k_{uagi}-M_{gilumen}\times(CLE_{fgi}+k_{giab})+M_{l}\times CLE_{fl}$ S5

where

*CLE_fgi_* [per hour] – Clearance rate from the GI tract to feces.

*k_giab_* [per hour] – Absorption rate of nanoparticles from the GI tract lumen to the blood in GI tract.

*M_l_* [µg] – Amount of nanoparticles in the liver.

*CLE_fl_* [per hour] – Clearance rate from the liver to feces.

Note the first two terms have a delay of 1.88 hours (fitted value) for the nanoparticles to come from the mouth to the GI tract lumen.

S2.2 Dynamics of the nanoparticles in tissues, as a function of capillary blood concentrations

The dynamic of nanoparticles in the tissue sub-compartment in different organs can be summarized as follow. The source of nanoparticles for tissues is capillary blood which is part of the systemic circulation. Parts of the nanoparticles will be exchanged back to capillary blood and return to the systemic circulation while other parts are captured by PCs. For those nanoparticles captured by PCs, desorption is also possible to re-enter the tissue. In certain organs, excretion out of the organ is a clearance route and/or transfer from the respiratory system contribute to the amount in these organs. The equation describing these processes is:

$\frac{dM_{t}}{dt}=\overbrace{PA_{t}\times(C_{t,b}-C_{t}/P)}^{\text{net transfer with capillary blood}}-\overbrace{(W_{t}\times C_{t}\times k_{t,ab}-M_{t,m}\times k_{de})}^{\text{interaction with PCs}}-\overbrace{\frac{dM_{e,ex}}{dt}}^{\text{clearance by excretion}}$ S6

where

*M_t_* [µg] – Amount of nanoparticles in the tissue of organ *t*.

*W_t_* [g] – Weight of organ *t*.

*C_t_* [µg per g] – Concentration of nanoparticles in the tissue of organ *t*.

*C_t,b_* [µg per g] – Concentration of nanoparticles in the capillary blood of organ *t*.

*PA_t_* [mL per hour] – Permeability coefficient-surface area cross-product. It is approximated as the product of the permeability coefficient between the capillary blood and tissue and regional blood flow, *χ_α_*×*Q_t_*, in this model, assuming the surface area is proportional to the regional blood flow.

*P* [unitless] – Partition coefficient of nanoparticles between tissue and blood.

*M_t,m_* [µg] – Amount of nanoparticles captured by PCs in organ *t*.

*k_t,ab_* [per hour] – Current uptake rate of nanoparticles by PCs in organ *t.*

*k_de_* [per hour] – Desorption rate of nanoparticles from PCs to tissue.

*M_e,ex_* [µg] – Amount to excreta from source *e*. *e* only applies to tissue in the liver and capillary blood in the kidneys. The elimination of nanoparticles directly from the GI tract is described separately

S2.3 Dynamics of the nanoparticles in the capillary blood, as a function of concentrations in the arterial and venous blood

The nanoparticle concentration in capillary blood is determined by the concentrations in the arterial blood and in the tissue. The interaction between capillary blood and tissue can be described as:

$\frac{dM_{t,b}}{dt}=Q_{t}\times(C_{art}-C_{t,b})-PA_{t}\times(C_{t,b}-C_{t}/P)$ S7

where

*M_t,b_* [µg] – Amount of nanoparticles in the capillary blood of organ *t*.

*Q_t_* [mL per hour] – Blood flow through organ *t*.

*C_art_* [µg per g] – Concentration of nanoparticles in arterial blood.

*C_t_* [µg per g] – Concentration of nanoparticles in the tissue of organ *t.*

Tissue uptake of nanoparticles from the capillary blood is modeled as a diffusion limited process, therefore the movement of the nanoparticles from capillary blood to the cellular matrix of the tissue is proportional to the permeability coefficient-surface area cross-product [2] which can be expressed as a proportion of the blood flow *Q_t_*, assuming the surface area is proportional to the regional blood flow:

$PA_{t}=\chi_{\alpha}\times Q_{t}$ S8

where *χ_α_* is the permeability coefficient between capillary blood and organ *t*. This parameter has a specific value for the brain (*χ_b_*) and the same generic value for the richly perfused organs (*χ_rich_*), which are the lungs, spleen, liver, kidneys, heart, and the GI tract.

We can simplify the model by eliminating the specific sub-compartment of the capillary blood and calculate concentration of the capillary blood as a function of the arterial blood, assuming $C_{t,b}$is in quasi steady state equilibrium between *C_art_* and *C_t_*. We then have:

$Q_{t}\times(C_{art}-C_{t,b})=PA_{t}\times(C_{t,b}-C_{t}/P)$ S9

Combining Eq. S8 and Eq. S9 we have the concentration in the organ capillary blood *C_t,b_* as:

$C_{t,b}=\frac{C_{art}+\chi_{\alpha}\times C_{t}/P}{(1+\chi_{\alpha})}$ S10

Eq. S10 applies to the heart, brain, kidneys, and the rest of the body in the systemic circulation. The capillary blood in the liver, spleen, and GI tract are described using Eq. S7 since they are locally interconnected via the portal vein. Because the lungs receive the collective venous blood, we have the concentration in the capillary blood of the lungs *C_lu,b_* as a function of the venous blood:

$C_{lu,b}=\frac{C_{ven}+\chi_{rich}\times C_{lu}/P}{(1+\chi_{rich})}$ S11

where

*C_ven_* [µg per g] – Concentration of nanoparticles in the venous blood.

*χ_rich_* [mL per hour] – Permeability coefficient between the capillary blood and the richly perfused organs.

*S2.4 Dynamics of the nanoparticles in arterial and venous blood*

For the arterial blood we have:

$\frac{dM_{art}}{dt}=Q_{tot}\times(C_{lu,b}-C_{art})-(W_{art}\times C_{art}\times k_{blood,ab}-M_{art,m}\times k_{de})$ S12

where

*M_art_* [µg] – Amount of nanoparticles in the arterial blood.

*Q_tot_* [mL per hour] – Total cardiac output.

*W_art_* [µg] – Weight of arterial blood.

*k_blood,ab_* [per hour] – Uptake rate of PCs in blood (mathematical description found later in this section)*.*

*M_art,m_* [µg] – Amount of nanoparticles captured by PCs in the arterial blood.

*k_de_* [per hour] – Desorption rate from PCs.

And the concentration of nanoparticles in the arterial blood *C_art_* is:

$C_{art}=\frac{M_{art}}{W_{art}}$ S13

The dynamic of nanoparticles in the venous blood is:

$\frac{dM_{ven}}{dt}=\sum(Q_{t}\times C_{t,b})-Q_{tot}\times C_{ven}-(W_{ven}\times C_{art}\times k_{blood,ab}-M_{ven,m}\times k_{de})$ S14

where

*M_ven_* [µg] – Amount of nanoparticles in the venous blood.

*C_ven_* [µg per g] – Concentration of nanoparticles in the venous blood*.*

*W_ven_* [g] – Weight of venous blood.

*M_ven,m_* [µg] – Amount of nanoparticles captured by PCs in the venous blood.

The concentration of nanoparticles in the venous blood *C_ven_* is:

$C_{ven}=\frac{M_{ven}}{W_{ven}}$ S15

Arterial blood is approximately 20% of the total blood and venous blood is approximately 80% of the total blood [3]. We also assume the PCs in the arterial and the venous blood are distributed in the same 20/80 mass ratio. Therefore, *W_art_* = 0.2 × *W_blood_*, *M_art,m_* = 0.2 × *M_blood,m_*, *W_ven_* = 0.8 × *W_blood_*, *M_ven,m_* = 0.8 × *M_blood,m_*, where *W_blood_* is the weight of the total blood and *M_blood,m_* is the amount of nanoparticles captured by PCs in the total blood.

S2.5 Dynamics of the nanoparticles in tissues, as a direct function of the arterial and venous concentrations

By introducing Eq. S10 to Eq. S6 we can directly express the dynamic amount of nanoparticles in the different organs (except for the lungs, liver, spleen, and GI tract) other than the blood as a function of the arterial blood, eliminating the sub-compartment of the capillary blood and simplifying the model as follows:

$\frac{dM_{t}}{dt}=\frac{\chi_{\alpha}\times Q_{t}}{(1+\chi_{\alpha})}(C_{art}-C_{t}/P)-(W_{t}\times C_{t}\times k_{t,ab}-M_{t,m}\times k_{de})-\frac{dM_{e,ex}}{dt}$ S16 The amount of nanoparticles in the interstitium of the lungs is related to the venous blood instead of the arterial blood and can be described as:

$\frac{dM_{lu}}{dt}=\frac{\chi_{r\text{est}}\times Q_{tot}}{(1+\chi_{rest})}(C_{ven}-C_{lu}/P)-M_{lu}\times k_{luip}+M_{lup}\times k_{lupi}-(W_{lu}\times C_{lu}\times k_{lu,ab}-M_{lu,m}\times k_{de})$ S17

where

*M_lu_* [µg] – Amount of nanoparticles in the lung tissue.

*W_lu_* [g] – Weight of the lungs.

*C_lu_* [µg per g] – Concentration of nanoparticles in the lungs.

*M_lu,m_* [µg] – Amount of nanoparticles captured by PCs in the lungs.

*k_lu,ab_* [per hour] – Current uptake rate of nanoparticles by PCs in the lungs*.*

In addition, the outflows from the spleen and GI tract connect directly to the liver, instead of joining the venous blood in circulation.

S2.6 Capture of nanoparticles by phagocytizing cells

The PCs capture nanoparticles from the tissue and in the meanwhile, exocytosis from the PCs will release some of the captured nanoparticles back to the tissue. The PCs capture the nanoparticles in a saturable manner. That is, as the amount captured approaches the capacity for the PCs in the various organs, the uptake rate *k_t,ab_* will decrease and eventually the capture of nanoparticles will be in a dynamic balance with the desorption. The equation describing these behaviors is:

$\frac{dM_{t,m}}{dt}=W_{t}\times C_{t}\times k_{t,ab}-M_{t,m}\times k_{de}$ S18

The initial uptake rate is assumed to be equal for all organs, while saturation levels of PCs will be adjusted for each compartment independently, reflecting the potential variation in the density of the PCs in various organs. Thus the uptake rate is related to the amount of nanoparticles already captured and the maximum uptake capacity in different organs:

$k_{t,ab}=k{}_{ab0}\times(1-\frac{M_{t,m}}{M_{t,cap}\times W_{t}})$ S19

where

*k_ab0_* [per hour] – Maximum uptake rate by PCs. The spleen has a separate maximum uptake rate due to its mesh-like structure.

*M_t,cap_* [µg] – PCs uptake capacity for nanoparticles per weight of organ *t*.

S2.7 Elimination

Some nanoparticles are assumed to be excreted by first order elimination from the GI tract lumen (either directly of the nanoparticles swallowed or via biliary excretion from the liver) and the capillary blood of the kidneys. This is summarized as below:

$\frac{dM_{ex}}{dt}=\sum\frac{dM_{e,ex}}{dt}=M_{gilumen}\times CLE_{fgi}+C_{art}\times CLE_{u}\times\frac{\chi_{rest}}{1+\chi_{rest}}$ S20

where

*M_ex_* [µg] – Amount in the excreta.

*CLE_u_* [per hour] – Clearance rate to urine from the capillary blood in the kidneys.

S2.8 Codes of the model

Codes of the model in Berkeley-Madonna^TM^ (version 8.3.18) are available by request to the corresponding author (Dingsheng Li, email: [dingsli@umich.edu](mailto:dingsli@umich.edu)).

*S2.9 References*

1. Stahl WR: **Scaling of Respiratory Variables in Mammals**. *J Appl Physiol* 1967, **22**:453–460.

2. Krishnan K: **Physiologically Pharmacokinetic and Toxicokinetic Models**. In *Principles and Methods of Toxicology*. Edited by Hayes A. Boca Raton: CRC press; 2007:231–292.

3. Despopoulos A, Silbernagl S: *Color Atlas of Physiology*. Thieme; 2003.
