# Supplementary material for: In vivo biodistribution and physiologically based pharmacokinetic modeling of inhaled fresh and aged cerium oxide nanoparticles in rats
Source: Part Fibre Toxicol. 2016 Aug 20;13:45. doi: 10.1186/s12989-016-0156-2 (PMC4992249; doi:10.1186/s12989-016-0156-2)
Supplement: Supplementary file 2 — Calculation of the deposition fractions in different regions of the respiratory system. Figure S1. Size distribution of the mass based concentration of CeO2 nanoparticles for four experiments based on the SMPS collected data. (DOCX 57 kb) [file 12989_2016_156_MOESM2_ESM.docx]

**Additional file 1 for**

*In vivo* biodistribution and physiologically based pharmacokinetic modeling of inhaled fresh and aged cerium oxide nanoparticles in rats

Calculation of deposition fractions in different regions of the respiratory system

We combined two sets of information to calculate the deposition fractions of nanoparticles in the upper airway, tracheobronchial region, and the pulmonary region. First we measured the detailed size distribution of nanoparticles in the study with the scanning mobility particle sizer (SMPS), the results of which are shown in Figure S1 below.

Fig. S1 Size distribution of the mass-based concentration of CeO_2_ nanoparticles for four experiments based on SMPS data.

Second, we utilized the Multiple-Path Particle Dosimetry Model (MPPD v2.11) to obtain the deposition fractions in different regions of the respiratory system for the size range of nanoparticles in this study, assuming the density of CeO_2_ nanoparticles in this study was 7.13 g/cm^3^. Then we matched the percentage of a certain size of nanoparticles in the study with the deposition fractions of the corresponding size in the MPPD model results. By repeating this process for the whole size distribution, we determined the cumulative deposition fractions of the inhaled nanoparticles for the different regions of the respiratory system. The results are shown in the following table, where Aged and Fresh refer to experiments that applied the aging chamber (Aged) and without the use of the aging chamber (Fresh).

| Parameter (unit) | Description | Fresh 1 | Fresh 2 | Aged 1 | Aged 2 |
| --- | --- | --- | --- | --- | --- |
| *fr_ua_* (unitless) | Fraction of inhaled nanoparticles deposited in the upper airway region | 0.098 | 0.11 | 0.096 | 0.097 |
| *fr_tra_* (unitless) | Fraction of inhaled nanoparticles deposited in the tracheobronochial region | 0.037 | 0.034 | 0.034 | 0.036 |
| *fr_pul_* (unitless) | Fraction of inhaled nanoparticles deposited in the pulmonary region | 0.16 | 0.14 | 0.14 | 0.16 |
