# Supplementary material for: In vivo biodistribution and physiologically based pharmacokinetic modeling of inhaled fresh and aged cerium oxide nanoparticles in rats
Source: Part Fibre Toxicol. 2016 Aug 20;13:45. doi: 10.1186/s12989-016-0156-2 (PMC4992249; doi:10.1186/s12989-016-0156-2)
Supplement: Supplementary file 3 — Concentrations found in feces in the pilot study. Error bars representing one standard deviation on the means. (DOCX 37 kb) [file 12989_2016_156_MOESM3_ESM.docx]

**Additional file 3 for**

*In vivo* biodistribution and physiologically based pharmacokinetic modeling of inhaled fresh and aged cerium oxide nanoparticles in rats

Time evolution of cerium concentration in feces after a single exposure to CeO_2_.

A pilot study was conducted to measure the evolution of CeO_2_ nanoparticle concentrations in the feces. Six rats were exposed to 770 ± 210 µg/m^3^ cerium oxide nanoparticles for 5 hours and feces were collected one, two, four, five, and seven days post exposure using metabolic cages. Exposure protocols are the same as described in Methods. The data show a peak fecal cerium concentration within the first 24 hour post exposure, and followed by a rapid decrease in until the fourth day, after which the cerium levels remained approximately the same.

Figure S2 Cerium concentration found in the feces in the pilot study. Error bars represent one standard deviation on the means.
