# Supplementary material for: In vivo biodistribution and physiologically based pharmacokinetic modeling of inhaled fresh and aged cerium oxide nanoparticles in rats
Source: Part Fibre Toxicol. 2016 Aug 20;13:45. doi: 10.1186/s12989-016-0156-2 (PMC4992249; doi:10.1186/s12989-016-0156-2)
Supplement: Supplementary file 4 — Individual organ concentrations of CeO2 nanoparticles for all experiments. (DOCX 39 kb) [file 12989_2016_156_MOESM4_ESM.docx]

**Additional file 4 for**

*In vivo* biodistribution and physiologically based pharmacokinetic modeling of inhaled fresh and aged cerium oxide nanoparticles in rats

Individual organ concentrations of CeO_2_ nanoparticles for all experiments

The following tables show the individual organ concentrations of CeO_2_ nanoparticles for all experiments. Rat 9-892 in experiment fresh 1, and rat 13-892 in experiment fresh 2 were deemed outliers due to exceptionally high concentrations of nanoparticles in the heart and feces respectively. These concentrations violated the mass balance by themselves and therefore the data from these two rats were discarded from further analysis of the data.

| **Experiment fresh 1** |  | | | | | | | | | | | | |  | |  | |  | |  | |  | |
| --- | --- | --- | --- | --- | --- | --- | --- | --- | --- | --- | --- | --- | --- | --- | --- | --- | --- | --- | --- | --- | --- | --- | --- |
|  | Exposure | Necropsy | Lung | | Liver | | Kidney | | Heart | | | Brain | | Spleen | | Olfactory | | Feces | | Urine | | Serum | |
| Rat ID | duration (hrs) | post exposure | (µg/kg) | | (µg/kg) | | (µg/kg) | | (µg/kg) | | | (µg/kg) | | (µg/kg) | | (µg/kg) | | (µg/kg) | | (ng/mL) | | (ng/mL) | |
| 1-892 | 4 | 15 min | 261 | | 0.6 | | 0.17 | | 0.10 | | | 0.17 | | 0.38 | | 0.8 | | - | | - | | 3.5 | |
| 2-892 | 4 | 15 min | 465 | | 0.4 | | 0.29 | | 0.14 | | | 0.16 | | 1.76 | | 0.7 | | - | | - | | 2.1 | |
| 3-892 | 4 | 15 min | 226 | | 0.4 | | 0.21 | | 0.14 | | | 0.16 | | 0.60 | | 0.6 | | - | | - | | 3.7 | |
| 4-892 | 4 | 24 hrs | 229 | | 1.7 | | 1.28 | | 0.16 | | | 0.13 | | 0.28 | | 0.9 | | 91 | | 1.9 | | 3.1 | |
| 5-892 | 4 | 24 hrs | 193 | | 1.2 | | 0.59 | | 0.15 | | | 0.69 | | 0.20 | | 0.8 | | 88 | | 7.4 | | 2.7 | |
| 6-892 | 4 | 24 hrs | 255 | | 1.5 | | 1.09 | | 0.81 | | | 15.83 | | 0.14 | | 0.8 | | 96 | | 8.1 | | - | |
| 7-892 | 4 | 7 days | 185 | | 9.9 | | 1.98 | | 0.29 | | | 0.69 | | 4.19 | | 0.9 | | - | | - | | 1.9 | |
| 8-892 | 4 | 7 days | 3 | | 3.4 | | 1.37 | | 0.37 | | | 0.85 | | 0.33 | | 0.5 | | - | | - | | 1.8 | |
| 9-892 | 4 | 7 days | 4 | | 0.1 | | 0.17 | | 1214.80 | | | 6.33 | | BID | | 87.2 | | - | | - | | 2.3 | |
| *BID: below detection limit | | |  | |  | |  | |  | | |  | |  | |  | |  | |  | |  | |
| - no sample | |  |  | |  | |  | |  | | |  | |  | |  | |  | |  | |  | |
|  | |  |  | |  | |  | |  | | |  | |  | |  | |  | |  | |  | |
| **Experiment fresh 2** |  | | | | | | | | | | | | |  | |  | |  | |  | |  | |
|  | Exposure | Necropsy | | Lung | | Liver | | Kidney | | Heart | Brain | | Spleen | | Olfactory | | Feces | | Urine | | Serum | |  |
| Rat ID | duration (hrs) | post exposure | | (µg/kg) | | (µg/kg) | | (µg/kg) | | (µg/kg) | (µg/kg) | | (µg/kg) | | (µg/kg) | | (µg/kg) | | (ng/mL) | | (ng/mL) | |  |
| 10-892 | 6 | 15 min | | 1645 | | 0.9 | | 0.92 | | 0.76 | 1.23 | | 0.15 | | 7.7 | | - | | - | | 2.3 | |  |
| 11-892 | 6 | 15 min | | 1431 | | 1.2 | | 0.98 | | 0.58 | 1.19 | | 0.15 | | 25.2 | | - | | - | | 2.7 | |  |
| 12-892 | 6 | 15 min | | 1704 | | 0.8 | | 0.94 | | 0.49 | 0.92 | | BID | | 6.2 | | - | | - | | 2.0 | |  |
| 13-892 | 6 | 24 hrs | | 1540 | | 2.3 | | 1.57 | | 0.53 | 0.97 | | 0.23 | | 3.1 | | 9654 | | - | | 2.1 | |  |
| 14-892 | 6 | 24 hrs | | 4 | | 0.5 | | 0.84 | | 0.47 | 0.93 | | 0.57 | | 10.0 | | 465 | | - | | 2.4 | |  |
| 15-892 | 6 | 24 hrs | | 1866 | | 2.6 | | 1.99 | | 0.65 | 0.96 | | 0.35 | | 4.6 | | 1074 | | - | | 2.2 | |  |
| 16-892 | 6 | 7 days | | 2017 | | 8.4 | | 5.74 | | 0.86 | 1.29 | | BID | | 5.9 | | - | | - | | 2.3 | |  |
| 17-892 | 6 | 7 days | | 13 | | 0.5 | | 1.20 | | 0.46 | 1.55 | | 6.24 | | 3.5 | | - | | - | | 1.9 | |  |
| *BID: below detection limit | | | |  | |  | |  | |  |  | |  | |  | |  | |  | |  | |  |
| - no sample  Rate 18-892 incurred mechanical stress and was removed from the study. | | | | | | | | | | | | | | | | | | | | | | |  |

| **Experiment aged 1** |  | | | | | | |  |  |  |  |  |  |
| --- | --- | --- | --- | --- | --- | --- | --- | --- | --- | --- | --- | --- | --- |
|  | Exposure | Necropsy | Lung | Liver | Kidney | Heart | Brain | Spleen | Olfactory | Feces | Urine | Serum | GI tract |
| Rat ID | duration (hrs) | post exposure | (µg/kg) | (µg/kg) | (µg/kg) | (µg/kg) | (µg/kg) | (µg/kg) | (µg/kg) | (µg/kg) | (ng/mL) | (ng/mL) | (µg/kg) |
| 1-893 | 4 | 15 min | 1136 | 0.55 | 0.25 | 0.11 | BID | 0.62 | BID | - | - | 1.4 | 89.8 |
| 2-893 | 4 | 15 min | 1754 | 0.19 | 0.31 | 0.16 | BID | 0.22 | BID | - | - | 1.4 | 70.0 |
| 3-893 | 4 | 15 min | 1031 | 0.18 | 0.15 | 0.47 | 0.34 | 0.18 | BID | - | - | 1.6 | 29.1 |
| 4-893 | 4 | 24 hrs | 507 | 1.32 | 0.48 | 0.81 | BID | 0.27 | BID | 235 | 3.1 | 1.5 | 27.6 |
| 5-893 | 4 | 24 hrs | 20 | 0.25 | 0.57 | BID | BID | 0.25 | BID | 686 | 0.9 | 2.0 | 2.1 |
| 6-893 | 4 | 24 hrs | 958 | 2.22 | 0.70 | 0.17 | BID | 0.30 | BID | 1096 | 1.0 | 1.8 | 14.4 |
| 7-893 | 4 | 7 days | 647 | 5.98 | 1.93 | 0.22 | BID | 0.41 | BID | - | - | 0.6 | 4.1 |
| 8-893 | 4 | 7 days | 1145 | 3.76 | 1.98 | 0.32 | BID | 0.46 | BID | - | - | 1.3 | 13.1 |
| 9-893 | 4 | 7 days | 533 | 9.34 | 1.26 | 0.20 | BID | 0.29 | BID | - | - | 0.9 | 12.2 |
| *BID: below detection limit | | |  |  |  |  |  |  |  |  |  |  |  |
| - no sample | |  |  |  |  |  |  |  |  |  |  |  |  |
| -- under analysis | |  |  |  |  |  |  |  |  |  |  |  |  |

| **Experiment aged 2** |  | | | | | | |  |  |  |  |  |  |
| --- | --- | --- | --- | --- | --- | --- | --- | --- | --- | --- | --- | --- | --- |
|  | Exposure | Necropsy | Lung | Liver | Kidney | Heart | Brain | Spleen | Olfactory | Feces | Urine | Serum | GI tract |
| Rat ID | duration (hrs) | post exposure | (µg/kg) | (µg/kg) | (µg/kg) | (µg/kg) | (µg/kg) | (µg/kg) | (µg/kg) | (µg/kg) | (ng/mL) | (ng/mL) | (µg/kg) |
| 10-893 | 4 | 15 min | 1289 | 0.43 | 1.38 | 1.50 | 2.12 | 1.28 | BID | - | - | 1.5 | 22.2 |
| 11-893 | 4 | 15 min | 1484 | 0.22 | 1.14 | 1.25 | 1.18 | 1.15 | BID | - | - | 1.0 | 32.3 |
| 12-893 | 4 | 15 min | 85 | BID | 1.07 | 1.32 | 1.61 | 1.15 | BID | - | - | 1.0 | 48.8 |
| 13-893 | 4 | 24 hrs | 1706 | 1.07 | 2.06 | 1.22 | 1.24 | 1.14 | 0.9 | 785 | 1.1 | 0.7 | 46.9 |
| 14-893 | 4 | 24 hrs | 1322 | 2.16 | 2.00 | 1.50 | 2.68 | 2.05 | BID | 630 | 4.3 | 0.5 | 19.3 |
| 15-893 | 4 | 24 hrs | 1236 | 1.68 | 1.72 | 1.26 | 1.55 | 1.46 | BID | 554 | 0.5 | 0.5 | 7.0 |
| 16-893 | 4 | 7 days | 571 | 2.98 | 2.53 | 1.43 | 1.08 | 2.17 | BID | - | - | 1.0 | 15.3 |
| 17-893 | 4 | 7 days | 1043 | 4.24 | 2.80 | 1.52 | 1.50 | 1.87 | BID | - | - | 0.5 | 8.6 |
| 18-893 | 4 | 7 days | 1174 | 3.50 | 3.56 | 1.45 | 1.63 | 1.71 | BID | - | - | 1.2 | 3.0 |
| *BID: below detection limit | | |  |  |  |  |  |  |  |  |  |  |  |
| - no sample | | |  |  |  |  |  |  |  |  |  |  |  |
| -- reanalysis | |  |  |  |  |  |  |  |  |  |  |  |  |
