# Supplementary material for: In vivo biodistribution and physiologically based pharmacokinetic modeling of inhaled fresh and aged cerium oxide nanoparticles in rats
Source: Part Fibre Toxicol. 2016 Aug 20;13:45. doi: 10.1186/s12989-016-0156-2 (PMC4992249; doi:10.1186/s12989-016-0156-2)
Supplement: Supplementary file 5 — Time history of nanoparticles in the individual organs for all experiments. (DOCX 122 kb) [file 12989_2016_156_MOESM5_ESM.docx]

**Additional file 5 for**

*In vivo* biodistribution and physiologically based pharmacokinetic modeling of inhaled fresh and aged cerium oxide nanoparticles in rats

Individual organ time histories simulated by the PBPK model

The following figures show the model predictions for the individual organs for each experiment compared with the measured data. The simulations results for the organs are further differentiated into PCs captured and the total cerium in the organ. The amount captured by the PCs in organ *t* and the total amount of nanoparticle mass in organ *t* are presented using the notation *Mtm* and *Mtt*, respectively. *Mgilumen* represents the amount attached on the GI tract lumen. *Mpult* represents the total amount in the pulmonary region. The symbols for the measured data are circles in the figures. The x-axis is time in hours, where 0 hour is the start of the exposure. The y-axis is the mass of nanoparticles, with the unit of µg. The numbers 1, 2, 3, and 4 at the end of each symbol indicate the conditions for the experiment and the simulation, where . 1: fresh 1; 2: fresh 2; 3: aged 1; 4: aged 2. Data are not presented for organs where the mass was below the detectable limit; only the model simulation results are shown. The feces data are not presented here as they are represented in Figure 5 of the paper and they do not have a PC sub-compartment. The order of figures for each experimental condition is: blood, spleen, liver, brain, heart, kidneys, lungs, and GI tract.

The results indicate the model predicts PCs capturing almost all of the nanoparticles in the organs. There are some free nanoparticles in the organs during the exposure period and shortly after, but the free nanoparticles decrease over time. One exception is the GI tract, where the model predicts most of the nanoparticle mass is attached to the lumen of the GI tract. The nanoparticles are not inside the GI tissue tissue nor captured by the PCs.

Experiment fresh 1

Figure S3.1 Time history of nanoparticles in the individual organs for experiment fresh 1.

Experiment fresh 2

Figure S3.2 Time history of nanoparticles in the individual organs for experiment fresh 2.

Experiment aged 1

Figure S3.3 Time history of nanoparticles in the individual organs for experiment aged 1.

Experiment aged 2

Figure S3.4 Time history of nanoparticles in the individual organs for experiment aged 2.
